# Phytoplankton of the Curonian Lagoon as a New Interesting Source for Bioactive Natural Products. Special Impact on Cyanobacterial Metabolites

**DOI:** 10.3390/biom11081139

**Published:** 2021-08-02

**Authors:** Donata Overlingė, Anna Toruńska-Sitarz, Marta Cegłowska, Agata Błaszczyk, Karolina Szubert, Renata Pilkaitytė, Hanna Mazur-Marzec

**Affiliations:** 1 Marine Research Institute, Klaipeda University, University Avenue 17, 92295 Klaipeda, Lithuania; renata.pilkaityte@apc.ku.lt; 2 Division of Marine Biotechnology, Faculty of Oceanography and Geography, University of Gdańsk, Marszałka J. Piłsudskiego 46, PL-81378 Gdynia, Poland; anna.torunska@ug.edu.pl (A.T.-S.); agata.blaszczyk@ug.edu.pl (A.B.); karolina.szubert@phdstud.ug.edu.pl (K.S.); hanna.mazur-marzec@ug.edu.pl (H.M.-M.); 3 Institute of Oceanology, Polish Academy of Sciences, Powstańców Warszawy 55, PL-81712 Sopot, Poland; mceglowska@iopan.pl

**Keywords:** phytoplankton, cyanobacteria, antibacterial compounds, enzymatic activity, cytotoxicity, acute toxicity, Baltic Sea

## Abstract

The bioprospecting of marine and brackish water systems has increased during the last decades. In this respect, microalgae, including cyanobacteria, and their metabolites are one of the most widely explored resources. Most of the bioactive compounds are isolated from ex situ cultures of microorganisms; however, analysis of field samples could also supply valuable information about the metabolic and biotechnological potential of microalgae communities. In this work, the activity of phytoplankton samples from the Curonian Lagoon was studied. The samples were active against antibiotic resistant clinical and environmental bacterial strains as well as against serine proteases and T47D human breast adenocarcinoma cells. No significant effect was found on *Daphnia magna*. In addition, using LC-MS/MS, we documented the diversity of metabolites present in field samples. A list of 117 detected cyanopeptides was presented. Cyanopeptolins constituted the largest class of cyanopeptides. As complex bloom samples were analyzed, no link between the observed activity and a specific sample component can be established. However, the results of the study showed a biotechnological potential of natural products from the Curonian Lagoon.

## 1. Introduction

The brackish water Curonian Lagoon, located along the south-eastern part of the Baltic Sea, is one of the largest lagoons in Europe. As a highly eutrophic water body, it annually experiences massive blooms of microalgae [1,2,3,4]. The spring phytoplankton community is dominated by diatoms, mainly *Stephanodiscus hantzschii*, *Diatoma tenuis*, *Aulacoseira islandica*, *Asterionella formosa* [5,6]. During the summer–autumn seasons, cyanobacteria are the main phytoplankton component. Among them, *Aphanizomenon* spp., *Planktothrix agardhii*, *Dolichospermum* spp., *Microcystis* spp., *Woronichinia compacta*, *Limnothrix redekei* are dominant [1,7,8]. Cyanobacteria and eukaryotic microalgae, mainly diatoms and green algae, generate a high diversity of metabolites, with an important ecological function and/or potential of biotechnological application.

Previous research in the Curonian Lagoon has only focused on the ecotoxicological assessment of cyanobacterial scum and cyanobacterial toxins from the perspective of the ecosystem and public health [7,9,10,11,12,13]. To our knowledge, no published data on the biological activity of metabolites produced by cyanobacteria and eukaryotic microalgae occurring in this lagoon exist. In terms of cyanobacterial toxins, the presence of various microcystin (MC) analogues, anatoxin-a (ANTX-a) and nodularin (NOD) has been confirmed [7,9,12,13]. Reports on the detection of cyanopeptides in the Curonian Lagoon was also published [10,13].

It is well documented that cyanobacteria are leaders among the natural sources of bioactive compounds. Most frequently, their cytotoxic effect has been described [14,15]. Like other microalgae groups (diatoms, green microalgae), cyanobacteria also show considerable antibacterial potential and can inhibit the growth of multidrug-resistant pathogens [16,17,18]. In addition, secondary metabolites produced by microalgae and cyanobacteria exhibit anti-inflammatory, antioxidant, anticoagulant, antiprotozoal and antiviral activities [19].

In general, the bioprospecting of marine and brackish water systems has highly increased during the last few decades. In this respect, microalgae and their metabolites are one of the most widely explored resources. Especially in areas such as pharmacy, aquaculture, bioremediation, bioenergy, biorefinery and biopigmentation [20,21]. Due to the urgent need for more effective and safer medicines for the treatment of cancer, metabolic disorders and infections caused by multidrug-resistant microorganisms, the search for new natural products for the pharmaceutical industry has been recently intensified [22,23,24].

To date, the potential for biotechnological application of more than 10,000 new compounds from these resources has been assessed [21,25]. Most of the bioactive compounds were isolated from ex situ cultures of microorganisms. However, in some cases, the biosynthesis of specific metabolites might not be triggered under laboratory conditions [17,26]. Some microalgae that live naturally in the aquatic environment show metabolic plasticity under stressed vs. non-stressed conditions. Abiotic and biotic stress can provide an extra advantage of triggering the synthesis of secondary metabolites [27]. However, the identification of their producer in field samples can present a challenge and the reproducibility of biological effects is rather unlikely. Therefore, in further studies isolated strains and their metabolites should be explored. On the other hand, the analysis of phytoplankton bloom samples supplies valuable information about the metabolic and biotechnological potential of the organisms living in the analyzed ecosystem [28].

The objectives of this study were: (1) to assess the biotechnological potential of phytoplankton from the Curonian Lagoon using enzymatic, antimicrobial, and cytotoxicity assays as well as acute toxicity assay; (2) to document the diversity of bioactive cyanometabolites from the Curonian Lagoon.

## 2. Materials and Methods

### 2.1. Field Samples Collection

**Figure 1:**
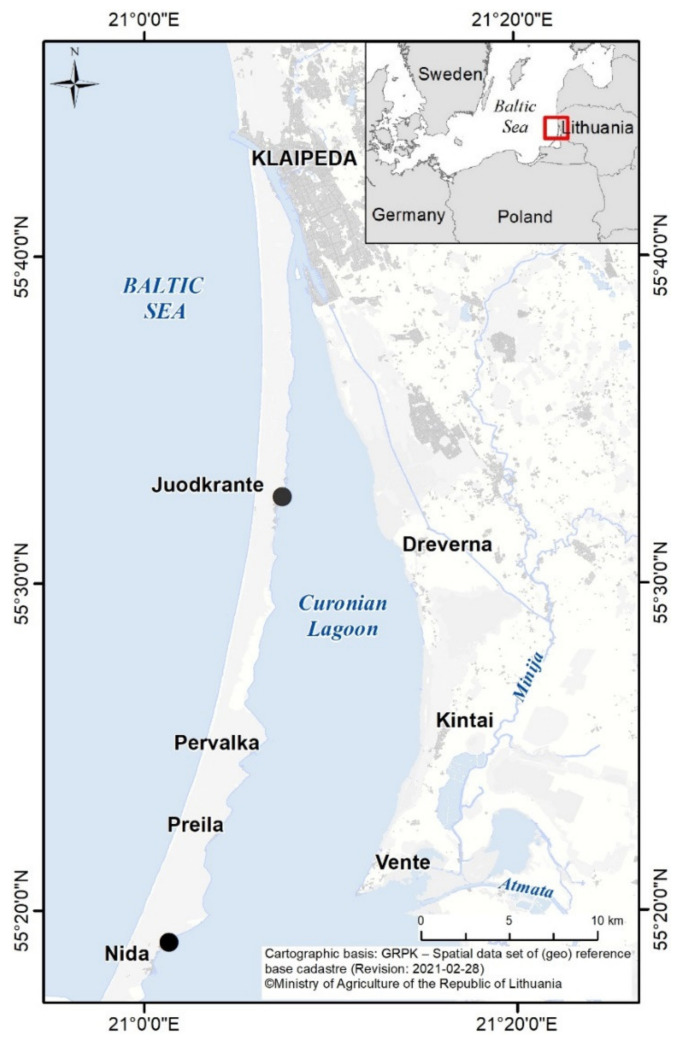
The study area and the locations (black circles) of the two sampling sites in the Curonian Lagoon (Nida and Juodkrante).

The samples (Sample ID 1–9, Table 1) were collected from two stations (Nida and Juodkrante (Figure 1), depending on the highest surface accumulation of the phytoplankton) every second week from June until August in 2018. To collect higher biomass for activity screening, water samples were concentrated using 55 µm Apstein plankton net and centrifuged at 3444× *g*, 8 °C for 10 min (Centrifuge 5810 R, Eppendorf^®^, Hamburg, Germany). Then, the samples were frozen and freeze-dried. Subsamples for phytoplankton analyzes, both concentrated and non-concentrated, were fixed with Lugol’s iodine solution.

### 2.2. Phytoplankton Analysis

The qualitative and quantitative analyses of the phytoplankton community composition were conducted using a LEICA DMI 3000 (Leica Microsystems CMS, Wetzlar, Germany) inverted microscope at magnifications of ×100 and ×400. The qualitative analysis of concentrated phytoplankton samples was carried out using Nunclon 10 mL 6-well chambers [28]. The quantitative phytoplankton analysis was performed according to the methodology described by Utermöhl [29]; the phytoplankton abundance and biomass was calculated according to the methodology described by HELCOM [30] and Olenina et al. [31]. Detailed description of the methods used for calculation of phytoplankton abundance and biomass can be found in the Supplementary Materials. Phytoplankton was identified to the lowest possible taxonomic level using guidelines described in the literature for the freshwater and brackish environments [32,33,34,35].

### 2.3. Extraction and Fractionation of Phytoplankton Biomass

Freeze-dried phytoplankton biomass was extracted with 75% methanol by vortexing for 15 min and centrifuged (19,837× *g*, 4 °C for 20 min) (Centrifuge 5810 R, Eppendorf^®^, Hamburg, Germany). The supernatants were diluted with MilliQ water, to lower the concentration of methanol below 10%. Then, the samples were partially purified by passing through preconditioned Waters Sep-Pak^®^ Vac 20cc C18 cartridges (5 g) (Waters, Milford, MA, USA). The extracts were eluted by washing the cartridges with 90% methanol in MilliQ water. The collected extracts were then evaporated using a miVac QUATTRO centrifugal vacuum concentrator (SP Scientific, Ipswich, UK) to the dry residue and depending on the bioassay (antibacterial, enzyme inhibition, cytotoxicity, or acute toxicity) (see methodology below) prepared for the first testing step (1st t.s.) (Table 1).

Based on the bioactivity response obtained in the MTT, antibacterial and enzyme inhibition assays (1st t.s.), extracts IV, V, VIII and IX, were selected for further investigation (Table 1, second testing step (2nd t.s.)). The dried extracts were dissolved in 75% methanol (10 mL) by vortexing for 10 min and diluted in MilliQ water to lower the methanol concentration (<10%), centrifuged (19,837× *g*, 4 °C for 10 min) (Centrifuge 5810 R, Eppendorf^®^, Hamburg, Germany) and filtered through GF/A filters (Whatman International Ltd., Kent, UK). The supernatants were then loaded onto a preconditioned flash chromatography Biotage^®^ SNAP KP-C18-HS (120 g) column (Biotage, Uppsala, Sweden). Fractionation was performed using Shimadzu HPLC system model LC-20AP (Shimadzu, Canby, OR, USA) equipped with a photodiode array detector (PDA). PDA operated in a range from 190 to 500 nm and during all chromatographic runs, the absorbance at 210 and 280 nm was recorded. Samples were loaded onto a preconditioned column at a flow rate of 12 mL min^−1^. After washing the resin with MilliQ water the sorbed substances were eluted with methanol: water mixture, gradually increasing the strength of the eluent (by 10% at each step) from 10% to 100% methanol. A volume of 12 mL was collected for each fraction. Based on the results of the MTT assay (2nd t.s.), fractions V-60 and V-70 were further separated (3rd t.s.)). The second (II) fractionation of V-60 and V-70 was performed manually using Waters Sep-Pak^®^ Vac (50 mg) (Waters, Milford, MA, USA). Sorbed metabolites were eluted with 20%, 40%, 60%, 90%, and 100% methanol in MilliQ water. The collected fractions (I and II fractionation) were evaporated as described above.

### 2.4. Antibacterial Activity

#### 2.4.1. Bacterial Strains

Antibacterial activity was tested against 8 bacterial strains—*Staphylococcus aureus* CCNPB/1505, *Pseudomonas aeruginosa* CCNPB/MBL, *Acinetobacter baumanii* CCNPB/O, *Enterococcus faecium* 45, *Aeromonas salmonicida* 2013, *Vibrio cholerae* 2329, *Vibrio diazotrophicus* Cd1 and *Klebsiella pneumoniae* CCNPB/1404 (Table S1). Four clinical isolates (no. 1–4) were kindly provided by Kamila Korzekwa PhD, Medical Laboratories Center Dialab (Wrocław, Poland) and are now kept in the Culture Collection of Northern Poland at Division of Marine Biotechnology (CCNP), University of Gdańsk. Environmental isolates were obtained from Ewa Kotlarska PhD, Institute of Oceanology Polish Academy of Sciences (isolates no. 5–7 from IO PAN MB Strain Collection Institute of Oceanology, Polish Academy of Sciences, Molecular Biology Laboratory) and Aneta Łuczkiewicz PhD, Gdańsk University of Technology (isolate no. 8 from Department of Water and Wastewater Technology Strain Collection) [36,37,38].

#### 2.4.2. Antibacterial Assay

Broth microdilution assay was performed according to the European Committee on Antimicrobial Susceptibility Testing (EUCAST) recommendations (http://www.eucast.org, accessed on 18 January 2021). Before the experiments, the bacteria were grown overnight on Mueller Hinton (Sigma-Aldrich, Steinheim, Germany) agar at 36 °C. For the experiment, bacterial suspensions (prepared in Ringer’s solution and equal to 0.5 of McFarland standard (10^8^ CFU mL^−1^)) were diluted to obtain ~1 × 10^6^ CFU mL^−1^. The assay was performed in 96-well sterile microplates (Eppendorf, Hamburg, Germany). The extracts and fractions were diluted in 2% v/v DMSO and tested in triplicates at concentrations of 500, 250, 100 and 10 μg mL^−1^. The fractions were prepared using a two-fold serial microdilution method. The concentration of the fractions used in the experiment ranged from 1.95 µg mL^−1^ to 1000 µg mL^−1^. The 96-well microplates were incubated overnight (Gram− for 16 h, Gram+ for 24 h) at 36 °C. The optical density (OP) of each well was measured at 620 nm using SpectraMax^®^ i3 Platform (Molecular Devices, San Jose, CA, USA). The percentage of growth inhibition was calculated in comparison to the control (bacterial culture without the extract/fraction).

### 2.5. Cytotoxicity Assay

Cytotoxicity assay was performed on human breast adenocarcinoma cell line T47D (Merck KGaA, Darmstadt, Germany). The assay was performed based on the colorimetric MTT assay method described by Felczykowska [39]. T47D cells were plated at a concentration of 1 × 10^4^ cells per well of 96-well plate containing RPMI1640 (Carl Roth GmbH & Co. KG, Karlsruhe, Germany) medium supplemented with 10% fetal bovine serum (Merck KGaA, Darmstadt, Germany) and penicillin–streptomycin solution (50 U and 0.05 mg mL^−1^, respectively; Merck KGaA, Darmstadt, Germany) and allowed to attach overnight (24 h of incubation at 37 °C; in CO_2_ (5%)). After the incubation, the medium was replaced with a fresh portion of extracts or fractions dissolved in 1% v/v DMSO (Merck KGaA, Darmstadt, Germany) at final concentrations 200, 100, 50 and 25 µg mL^−1^. The test was performed in three replicates for all the tested extracts, fractions and control samples. Plates were incubated for 24 h (37 °C). Then, 25 µL of MTT solution (4 mg mL^−1^) was added to each well. After 4 h of incubation, the medium was removed and 100 µL of 100% DMSO was added to dissolve the formazan. The absorbance of the reaction mixtures was measured at 570 nm (with reference wavelength 660 nm) using a microplate reader (Spectramax i3, Molecular Devices, San Jose, CA, USA). Cell survival was calculated as the ratio of the mean absorbance of the tested samples in comparison to the control (mean absorbance of the corresponding solvent) and expressed as a percentage.

### 2.6. Enzyme Inhibition Assay

Trypsin inhibition assay was performed according to the methodology described by Pluotno and Carmeli [40], chymotrypsin and thrombin assay followed the procedure by Ocampo Bennet [41]. The extracts and fractions were diluted in 1% v/v DMSO at final concentrations of 45 and 4.5 µg mL^−1^. Standard inhibitors were used as a positive control and 1% v/v DMSO with the addition of buffer, as a negative control. The absorbance of the solutions was measured at 405 nm with the application of a microplate reader (Varioskan Flash Thermo Fisher Scientific OY, Finland).

The final concentrations of enzymes used for the assays were 0.1 mg mL^−1^ for trypsin and chymotrypsin and 0.5 mg mL^−1^ for thrombin. In the case of trypsin and chymotrypsin, the mixtures containing the sample (10 µL) or inhibitor (10 µL), enzyme (10 µL) and buffer (100 µL) were preincubated for 5 min at 37 °C. Then, the substrate solution (100 µL) was added, and the mixture was incubated for 10 additional min at 37 °C. In the case of thrombin, the sample (10 µL) or inhibitor (10 µL), with the addition of enzyme (10 µL) and buffer (170 µL) were preincubated at 36 °C for 10 min after which 20 µL of the substrate was added. The solution was incubated for another 10 min at 36 °C. The percentage of enzymatic inhibition was calculated in comparison to the positive control.

### 2.7. Acute Toxicity Assay

The toxicity of phytoplankton extracts towards the juvenile freshwater cladoceran *Daphnia magna* was evaluated in 24-h and 48-h bioassays. The tests were performed according to the procedure described by the producer (MicroBioTests Inc., Gent, Belgium) protocol. The test organisms were prepared for the experiment by incubating their cryptobiotic forms in Standard Medium (SM). The extracts were dissolved in 1% DMSO at final concentrations 10, 5 and 2.5 µg mL^−1^. Specimens of *D. magna* were exposed to 10 mL of the prepared extracts. The assay plates containing ephippia (5 in each well) were incubated at 20 °C, 6000 lux. The assay was performed in triplicate. The test endpoint was the death of the organisms. The results were presented as the percentage of surviving organisms.

### 2.8. Analysis of Cyanometabolites

Analyses of cyanometabolites were done using Agilent HPLC system (Agilent Technologies, Waldboronn, Germany) coupled to a hybrid triple quadrupole/linear ion trap mass spectrometer QTRAP LC-MS/MS (QTRAP5500, Applied Biosystems, Sciex; Canada) according to the method described by Mazur-Marzec et al. [42]. Chromatographic separation was performed on a Zorbax Eclipse XDB-C18 column (4.6 µm, 150 mm, 5 µm; Agilent Technologies, Santa Clara, CA, USA). To determine the content of crude extracts and fractions, the information-dependent acquisition method (non-target analysis) was used. Total ion current spectra were used to determine the most intense ion peaks. Data were processed with Analyst QS (Version 1.5.1, Applied Biosystems/MDS Analytical Technologies, Concord, ON, Canada, 2008).

### 2.9. Statistical Analysis

Non-parametric multidimensional scaling (nMDS) based on the Jaccard similarity coefficient [43] of presence-absence data, was used to represent the similarities of phytoplankton communities among the different samples. Phytoplankton samples were divided into groups with group-average linking [44]. One-way ANOSIM tests were used to determine the significances of the degree of separation among the nMDS groups. The stress values of the two MDS plots were determined, which is considered to adequately represent the similarity between the samples in nMDS plots. A stress value of <0.2 indicates an accurate representation of similarity rankings. nMDS and ANOSIM analyses were conducted using Primer v6 software. All biological experiments were carried out in triplicate, the data presented in this paper is expressed as a mean. The reliability of results was verified through the calculation of standard deviation.

## 3. Results

### 3.1. Phytoplankton Community

In total, 178 species were observed: 68 members of Chlorophyta, 35 of Bacillariophyta, 52 of Cyanophyta, 7 of Cryptophyta, 8 of Dinophyta, 3 of Euglenophyta, 2 of Chrysophyta and 1 of Haptophyta (Table S8). The phytoplankton species composition in the concentrated and non-concentrated samples did not differ. Single phytoplankton cell sizes varied from 0.4 to 700 μm, some were chain or colony-forming species.

**Figure 2:**
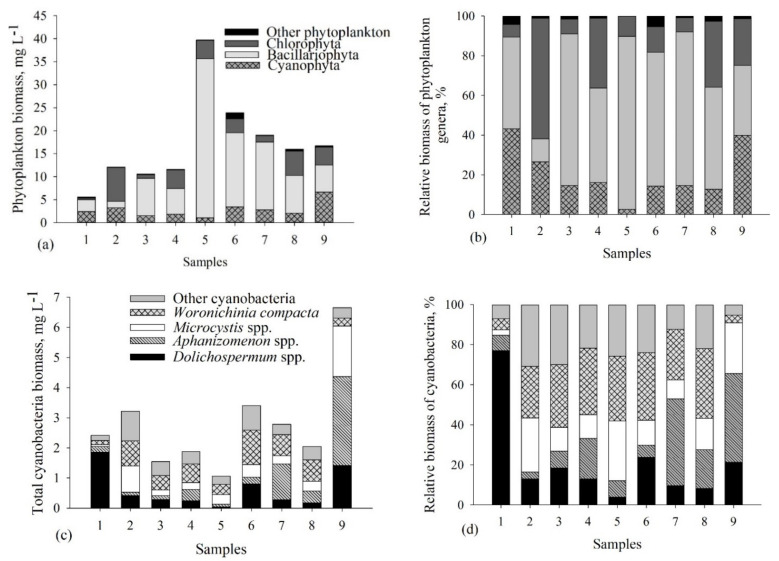
Structure, biomass (mg L^−1^) and relative biomass of phytoplankton (**a**,**b**) and cyanobacteria (**c**,**d**) community in the collected samples.

Phytoplankton biomass and dominating phytoplankton groups differed among the samples (Figure 2, Table S8). Bacillariophyta accounted for the highest biomass in the majority of the samples (Figure 2a,b). The highest total biomass of the Bacillariophyta was measured in Samples 5, 6 and 7 (67–87% of the total phytoplankton biomass (TPB)), the lowest was in Sample 2 (12% of the TPB). Amongst the diatoms, *Actinocyclus normanii* accounted for the highest contribution of the TPB (30–70% from the TPB), except Sample 2 (4% from the TPB) (Table S8). Chlorophyta dominated only in Sample 2 (61% of the TPB) (Figure 2a,b). Other phytoplankton groups did not exceed the 5% threshold of the TPB.

The highest contribution of cyanobacteria biomass was measured in Samples 1, 2 and 9 (27–43% of the TPB), the lowest was in Sample 5 (3% of the TPB) (Figure 2c,d, Table S8). *Dolichospermum* spp., *Aphanizomenon* spp., *Microcystis* spp. and *Woronichinia compacta* belonged to the dominant cyanobacterial genera (Figure 2c,d). An evident contribution of *Dolichospermum flosaquae* was observed only in Sample 1 (47% of the total cyanobacterial biomass (TCB)), while in other samples this species accounted for less than 7% of the TCB (Table S8). In other samples, different species of *Dolichospermum* were also present (*D. crassum*, *D. planctonicum*, *D. lemmermanii*) and accounted for not more than 11% of the TCB. In comparison, *Microcystis* genus, *M. wesenbergii* and *M. flosaquae*, had a higher contribution to the biomass of Samples 2, 5 and 9 (~10% of the TCB). The highest contribution of *Aphanizomenon flosaquae* was observed in Samples 7 and 9. *W. compacta* predominated in almost all samples (Samples 2–8) and accounted for 25–35% of the TCB (Figure 2c,d, Table S8).

In order to highlight the importance of different phytoplankton communities leading to the potentially different bioactivity results (Table 2 and Table 3, see results below), nMDS analysis of similarity was performed for each of the dominating phytoplankton communities (Bacillariophyta, Chlorophyta and Cyanophyta) separately (Figure S1). According to the obtained results based on Bacillariophyta and Chlorophyta, six samples out of nine did not differ in species composition and were grouped into one cluster (Group A) (Global R − 1, *p* < 0.01) (Figure S1a,b). Despite the similarity of species composition in Group A, samples showed different bioactivity results in antibacterial, enzymatic, cytotoxic and acute toxicity bioassays (Table 2 and Table 3). Considering the Cyanophyta community, nMDS analysis showed that the species composition was highly different among the samples (Global R − 0.765, *p* < 0.01) (Figure S1c) and indicated that the diversity of cyanobacteria influenced the recorded activity.

### 3.2. Bioactivity Screening of the Phytoplankton Extracts

To evaluate the biological activity of the extracts obtained from the collected phytoplankton, four different assays were applied (Table 2 and Table 3, Tables S3 and S4). Considering the antibacterial assay, at least 50% growth inhibition of the clinical strains (compared to the control) was obtained for the extracts II-VI (Table 2 and Table S3). In the case of *S. aureus*, extract IV, at 250 µg mL^−1^, reduced bacterial growth to less than 20% as compared to the control. Extract V, at 250 and 500 µg mL^−1^, inhibited two clinical strains, *S. aureus* and *P. aeruginosa* by more than 70% and 50%, respectively. The growth of the environmental strains was inhibited by all tested extracts, except for extract VIII. *V. diazotrophicus* and *A. salmonicida* were found to be most sensitive to phytoplankton extracts I − VII. None of the extracts reduced the growth of *V. cholerae* and even slight growth stimulation was observed for the extracts IV and V. Extract IX only inhibited the growth of *E. faecium* 45 (by 51%). Irrespective of the strain used in the tests, extract VIII did not show any antibacterial activity.

In the enzymatic assay, all tested extracts inhibited trypsin and thrombin at the highest concentration (45 µg mL^−1^) (Table 3 and Table S4). The strongest effect was exhibited by extracts VIII and IX, which inhibited trypsin and thrombin even at the lowest concentration applied in the assay (4.5 µg mL^−1^; >50%).

Considering the cytotoxicity assay, extracts II-VII decreased T47D cancer cells viability by more than 80% when applied at the highest concentration (200 µg mL^−1^). At 50 µg mL^−1^, extract IV was most active (Table 3 and Table S4).

In the acute toxicity assay, after 24-h exposure, the extracts did not decrease the survivorship of *D. magna* (Table 3 and Table S4). Only the 48-h assay revealed a toxic effect of the samples. The dilutions of the extract I decreased the survivorship of cladocerans by approximately 52% (applied at 2.5 µg mL^−1^) and 58% (applied at 5 µg mL^−1^), in comparison with untreated organisms. Other extracts had a lower toxicity effect on *D. magna*; in the tests, 60–90% of the organisms survived.

### 3.3. Bioactivity Screening of Fractions

The extracts with the highest activity revealed in the assays were chosen for further fractionation and analyses. Extracts IV and V were chosen for antibacterial assays, extract IV for cytotoxicity assays and extracts VIII and IX for enzymatic assays (Table 4). As the effects of the samples on *D. magna* were rather weak and did not exceed 40% of the control, further tests on this organism were not performed.

Fractions obtained from extracts IV and V inhibited the growth of four bacterial strains out of six applied in the antibacterial assays (Table 4 and Table S5). The fractions eluted with the solution containing the highest content of organic solvent (i.e., methanol) (IV-70 − 100, V-70 − 100) were highly active against the tested strains, mainly *S. aureus* CCNPB/1505 (bacterial growth reduced to less than 20% of control) and *V. cholerae* 2329 (reduced to less than 10%). The growth of *A. salmonicida* 2013 and *V. diazotrophicus* Cd1 was inhibited by almost all tested fractions (bacterial growth reduced to less than 50% of the control).

In the cytotoxicity assays, the viability of T45D cancer cells was affected by fractions IV-60 and IV-70 (Table 4 and Table S7). The most potent activity was observed for the fraction IV-70, which decreased cell viability to 47% of the control at the lowest concentration used in the assay (25 µg mL^−1^). This fraction was used for the third testing step and the effect was observed only for the subfraction IV-70-90 (cell viability reduced to 22% at 200 µg mL^−1^).

In enzyme inhibition assays, fractions 40-100 obtained from the extracts VIII and IX showed inhibitory activity against tested enzymes (Table 4 and Table S6). In the case of trypsin, chymotrypsin and thrombin, the highest inhibitory activity was obtained for fractions 40, 70 and 80 (more than 80% applied at 4.5 µg mL^−1^), fraction 70 (more than 60% applied at 4.5 µg mL^−1^) and fraction 50 (more than 60% applied at 4.5 µg mL^−1^), respectively.

### 3.4. Analysis of Cyanopeptides

In bioactive fractions obtained from extracts IV, V, VIII and IX, 117 cyanopeptides were detected (Table S2). Although in many cases only partial structure identification was possible, due to the presence of several diagnostic ions in the fragmentation spectra (Figure 3, Figure 4, Figure 5, Figure 6 and Figure 7, Figures S3–S12), the compounds could be assigned to one of the five cyanopeptide classes: the most numerous cyanopeptolins (CPs; 53 variants), microcystins (MCs; 19 variants), microginins (MGs; 18 variants), anabaenopeptins (APs; 14 variants) and aeruginosins (AERs; 13 variants).

**Figure 3:**
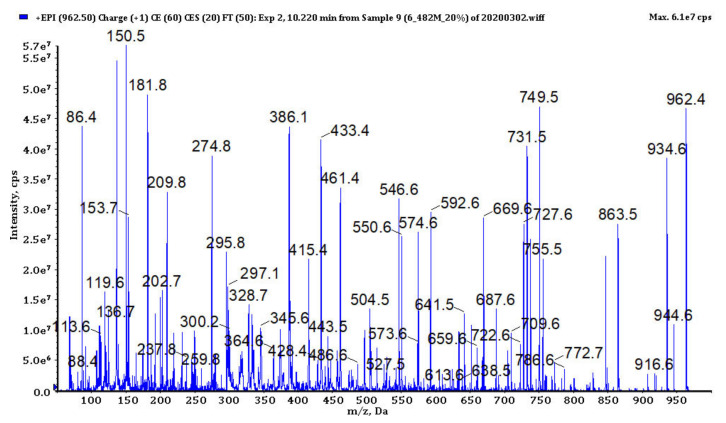
The enhanced product ion mass spectrum of cyanopeptoline CP979 with the suggested structure HA + Asp − [Thr^1^ + Tyr^2^ + Ahp^3^ + Ile^4^ + MeTyr^5^ + Val^6^] and the following fragment ions: *m*/*z* 962 [M + H − H_2_O]^+^, 944 [M + H − 2H_2_O]^+^, 934 [M + H − H_2_O − CO]^+^, 916 [M + H − 2H_2_O − CO]^+^, 863 [M + H − H_2_O − HA]^+^, 749 [M + H − H_2_O − (HA + Asp)]^+^, 731 [M + H − 2H_2_O − (HA + Asp)]^+^, 650 [M + H − H_2_O − (HA + Asp) − Val]^+^, 461 [HA + Asp + Thr + Tyr + H − H_2_O]^+^, 386 [Ahp + Ile + MeTyr + H − H_2_O]^+^, 297 [Asp + Thr + Val + H − H_2_O]^+^, 209 [Ahp + Ile + H − H_2_O]^+^, 181 [Ahp + Ile + H − H_2_O − CO]^+^, 150 MeTyr immonium, 136 Tyr immonium, 86 Ile immonium (HA − hexanoic acid, Ahp − 3-amino-6-hydroxy-2-piperidone).

The detection of CPs in the samples was based on spectral data published by Fuji et al. [45], Welker et al. [26,46] and Czarnecki et al. [47]. Structures of the peptides were mainly recognized by the ion peaks at *m*/*z* 420, 308, 234, 215, 150 (MeTyr immonium ion) and 120 (Phe immonium ion) that indicate the presence of Ahp + Phe + MeTyr fragment (Figure 3, Figures S3–S5; Ahp-3-amino-6-hydroxy-2-piperidone). In eight CPs (CP1012, CP993, CP986, CP984, CP965, CP951, CP911 and CP886) the substitution of chloride ion at MeTyr was detected by the shift of the peaks at *m*/*z* 420, 308 and 150 to *m*/*z* 454, 342 and 184. In few CPs spectra, the ion peaks at *m*/*z* 386, 274, 209, 181 and 86 that are characteristic for Ahp + Leu + MeTyr were observed. All Tyr^2^-containing CPs were detected as dehydrated protonated molecules [M + H − H_2_O]^+^ (Figure 3).

**Figure 4:**
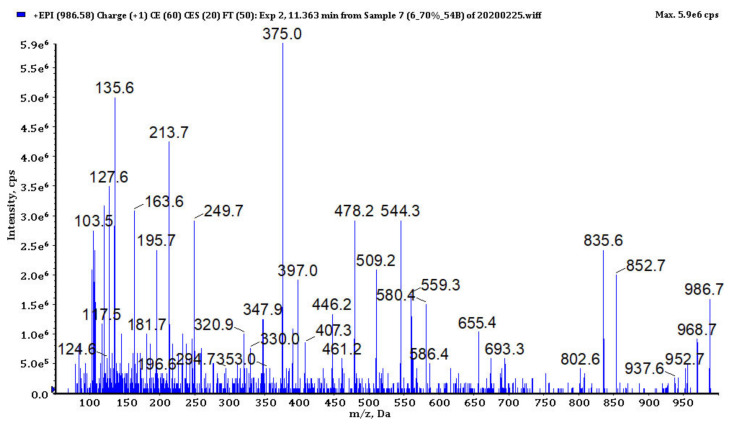
The enhanced product ion mass spectrum of microcystin MC-LF with the following fragment ions: *m*/*z* 986 [M + H]^+^, 968 [M + H − H_2_O]^+^, 802 [MeAsp + Phe + Adda + Glu + MeDha + H]^+^, 655 [M + H − Adda − H_2_O]^+^, 580 [Adda + Glu + MeDha + Ala + H − NH_3_]^+^, 559 [C_11_H_14_O + Glu + MeDha + Ala + Leu + H]^+^, 544 [MeDha + Ala + Leu + MeAsp + Phe + H]^+^, 509 [Adda + Glu + MeDha + H − NH_3_]^+^, 461 [Ala + Leu + MeAsp + Phe + H]^+^, 446 [C_11_H_14_O + Glu + MeDha + Ala + H]^+^, 407 [Leu + MeAsp + Phe + NH_4_]^+^, 397 [Glu + MeDha + Ala + Leu + H]^+^, 375 [C_11_H_14_O + Glu + MeDha + H]^+^, 213 [Glu + MeDha + H]^+^, 127 [MeDha + Ala + H − CO]^+^, 120 Phe immonium (Adda − (3-amino-9-methoxy-2,6,8-trimethyl-10- phenyldeca-4,6-dienoic acid) MeDha-methyldehydroalanine).

**Figure 5:**
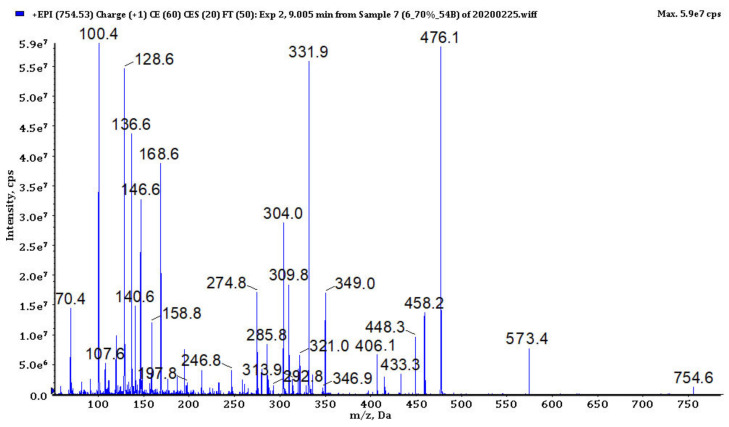
The enhanced product ion mass spectrum of microginin MG753 with the suggested structure Ahda + Tyr + MeIle/MeLeu + Pro + Tyr and the following fragment ions: *m*/*z* 754 [M + H]^+^, 573 [M + H − Tyr]^+^, 476 [M + H − (Tyr + Pro)]^+^, 458 [M + H − (Tyr + Pro) − H_2_O]^+^, 448 [M + H − (Tyr + Pro) − CO]^+^, 406 [MeLeu + Pro + Tyr + H]^+^, 349 [M + H − (Tyr + Pro + MeLeu)]^+^, 331 [M + H − (Tyr + Pro + MeLeu) − H_2_O]^+^, 321 [M + H − (Tyr + Pro + MeLeu) − CO]^+^, 279 [Pro + Tyr + H]^+^, 168 [Ahda − H_2_O]^+^, 158 [Ahda − CO]^+^, 136 Tyr immonium, 128 Ahda fragment, 100 MeLeu immonium, 70 Pro immonium (Ahda − 3-amino-2-hydroxy-decanoic acid).

**Figure 6:**
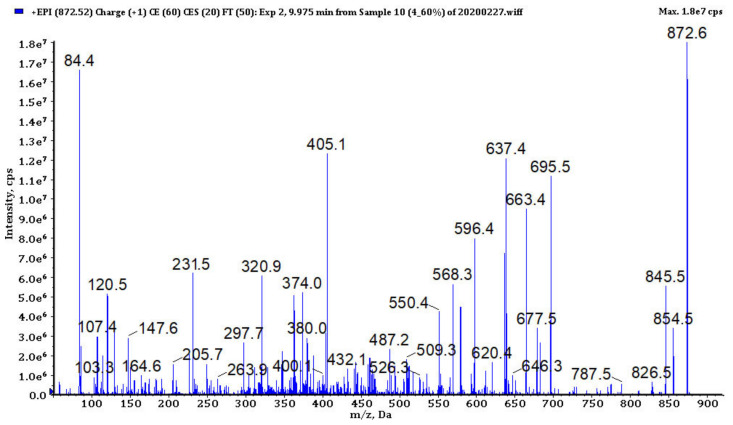
The enhanced product ion mass spectrum of anabaenopeptin AP871 with the suggested structure MeHTyr + CO − [Lys + Val + Hty + MeAla + Phe] and the following fragment ions: *m*/*z* 872 [M + H]^+^, 854 [M + H − H_2_O]^+^, 845 [M + H − CO]^+^, 826 [M + H − H_2_O − CO]^+^, 787 [M + H − MeAla]^+^, 773 [M + H − Val]^+^, 695 [M + H − HTyr]^+^, 663 [M + H − MeHTyr − H_2_O]^+^, 596 [M + H − (HTyr + Val)]^+^, 578 [M + H − (HTyr + Val) − H_2_O]^+^, 568 [M + H − (HTyr + Val) − CO]^+^, 550 [M + H − (HTyr + Val) − H_2_O − CO]^+^, 405 [HTyr + Val + Lys + H]^+^, 263 [MeAla + HTyr + H]^+^, 231 [MeAla + Phe − H]^+^, 164 MeHTyr immonium, 120 Phe immonium, 58 MeAla immonium, 84 Lys.

**Figure 7:**
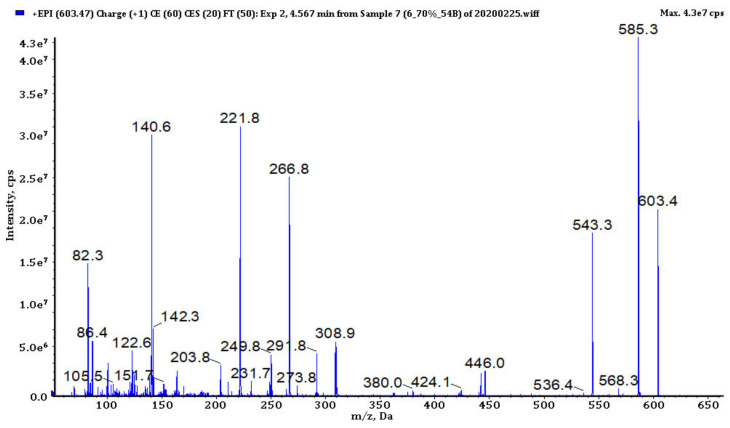
The enhanced product ion mass spectrum of aeruginosin K139 with the structure Hpla + Ile + Choi + Argal and the following fragment ions: *m*/*z* 603 [M + H]^+^, 585 [M + H − H_2_O]^+^, 543 [M + H − CH_2_N_2_ − H_2_O]^+^, 446 [M + H − Argal]^+^, 308 [Choi + Argal + H − NH_3_]^+^, 291 [Choi + Argal + H –NH_2_ − H_2_O]^+^, 266 [Choi + Argal + H − CH_3_N_2_ − H_2_O]^+^, 122 and 140 Choi ions, 142 Argal, 86 Leu immonium (Choi − 2-carboxy-6-hydroxyoctahydroindol, Hpla − (4-hydroxy)phenyllactic acid)).

The fragmentation spectra of MCs detected in samples from the Curonian Lagoon were compared with those published by other authors [48,49,50]. Based on the analysis (Figure 4, Figure S6) and the comparison of the spectra, we concluded that the detected MCs belong to the known MC variants previously described by Bouaïcha et al. [51] and included in CyanoMetDB [52]. Structure elucidation of MGs was mainly based on mass fragmentation spectra of the compounds published by Zervou et al. [53] and Carneiro et al. [54] and on the description of the important diagnostic ions of the peptides included in the work (e.g., *m*/*z* 128 for 3-amino-2-hydroxy-decanoic acid Ahda or *m*/*z* 142 for MeAhda). The process of structure elucidation of a new MGs variant, MG753, is illustrated in Figure 5 (Figure S7, Figure S8; MG928, MG783, respectively). In structure elucidation of APs, the fragmentation spectra published by Erhard et al. [55], Welker et al. [26] and Spoof et al. [56] were useful. The spectra always contained the peak at *m*/*z* 84 derived from the conserved Lys (Figure 6, Figures S9–S11). The kind of residue in a side chain was deduced based on the intensive ion peak at *m*/*z* [M + H − (Arg/Tyr/Ile − CO)]. The presence of Arg in this position was additionally confirmed by peaks at *m*/*z* 201 and 175. The MS/MS spectrum of AP871 and the description of the most characteristic fragment ions are shown in Figure 6. The fragmentation spectra of AERs were compared with those published by other authors [57] and was recognized by the presence of ions at *m*/*z* 140 and 122 characteristic for 2-carboxy-6-hydroxyoctahydroindole (Choi), and peaks at *m*/*z* 266, 291 and 308 indicating the presence of Choi + Argal fragment (Figure 7, Figure S12; 603, 619, respectively).

In the fractions eluted with the solution containing the highest content of organic solvent (i.e., methanol) (70–100) obtained from extracts IV and V, CPs and MGs constituted the dominant classes of peptides, with the highest number of detected variants, while the most intensive ion peaks in LC-MS chromatograms were observed for AERs and CPs (Table S9, Figures S2a–d). Of these, AER604 had the most intensive ion peak in the chromatograms of fractions IV-70 and V-70. In the fraction IV-80, only variant CP1006 was detected, while in the chromatogram of fraction V-90–CP1014 gave the most intensive ion peak. In the subfraction IV-70-90 the most intensive ion peaks in LC-MS chromatogram were observed for CPs, with CP1015 characterizes by the most intensive peak (Table S9, Figure S2e).

In the fractions (40–100) obtained from extracts VIII and IX, a high number of CPs variants were detected. High diversity of MGs was detected in the fractions VIII-80 and VIII-90, too. However, AERs, and especially AER567, gave the most intensive ion peaks in LC-MS chromatograms of the samples (Table S9, Figure S2f–j).

## 4. Discussion

Within this study, enzymatic, antimicrobial and cytotoxicity bioassays revealed high bioactivity of phytoplankton field samples collected from the Curonian Lagoon. Our results also showed that species diversity of phytoplankton, especially cyanobacteria, had a significant effect on the different bioactivity results. Bacillariophyta and Chlorophyta, two dominating phytoplankton groups did not differ in species diversity throughout the bioactive samples, while Cyanophyta species composition differed significantly. This fact suggests that secondary metabolites produced by cyanobacteria potentially may have had a greater influence on the bioactivity results than the compounds produced by eukaryotic microalgae. Unfortunately, as complex bloom samples were analyzed, no reliable conclusion about the link between the observed activity and a specific sample component can be established. As in the study, the LC-MS/MS method optimized for cyanopeptide analysis was used, numerous compounds from this group of natural products could be detected. A list of 117 cyanopeptides is presented in Table S2. CPs were found to be the dominant and one of the most structurally diverse class of cyanopeptides. Peptides representing other classes, i.e., AERs, APs, MGs, MCs, were 3–4-fold less numerous in different variants. The composition of cyanopeptides in the environment highly depends on the diversity of cyanobacteria species and their genetic ability to effectively biosynthesize these metabolites under various biotic and abiotic conditions [52,58,59,60].

During our study the ecological significance of phytoplankton metabolites was assessed using extracts and fractions obtained from field samples. The samples were tested with the application of different environmental bacterial strains and *D. magna*. The activity of extracts against *D. magna* was rather weak—even after 48 h of incubation more than 50% of the individuals survived (except extract I obtained from Sample 1). *Daphnia* sp. is an important organism in the food chains; it grazes on phytoplankton organisms [61]. In many acute toxicity assays, relatively high concentrations of samples are used, which do not always reflect the real situation in the aquatic environment. These conditions might potentially correspond periods of phytoplankton blooms or their final stages, when cells collapse, and concentrations of the dissolved secondary metabolites increases significantly. Such conditions could have a negative effect on aquatic organisms (including zooplankton) [62,63]. However, during non-bloom periods, zooplankton uses mechanisms that help to maintain persistent coexistence of both groups (cyanobacteria or microalgae and zooplankton). According to the literature, the zooplankton response to microalgae may vary between species or strains. Moreover, zooplankton has detoxification mechanisms to minimize the negative effects of cyanobacteria [64]. In our study the concentrations of extracts used for acute toxicity assay potentially reflected non-bloom conditions and it is possible that the defense mechanisms were effective enough for the organisms to support their survival.

In terms of the antibacterial assays, the growth of almost all environmental bacterial strains, except antibiotic-resistant *V. cholerae* 2329, was inhibited by the tested samples (Table 2). The tests revealed the ability of phytoplankton species to produce antibacterial compounds potently active against environmental, naturally occurring *A. salmonicida* and *V. diazotrophicus*. These compounds might constitute an element of a defense strategy and increase the survivorship of the producer in extremely competitive environments where a huge variety of bacteria and other microbes co-exists [28,65]. In the case of *V. cholerae* 2329, no inhibition or even growth stimulation was observed during our study. *Vibrio* are naturally occurring marine and brackish water bacteria and their intensive proliferation mostly correlates with temperature (>20 °C) and salinity (5–10 ppt) [66]. It is known that several species of *Vibrio* are pathogenic and may cause toxigenic cholera and vibriosis [67]. In natural water bodies, rising water temperature [68], which is one of the major causes of cyanobacterial blooms and proliferation [69,70], may also provide an optimal environment for the occurrence of *Vibrio* species [66,71]. Moreover, the dissolved organic matter resulting from intensive phytoplankton blooms, especially cyanobacteria-derived organic matter, can significantly support the growth of potentially pathogenic *Vibrio* species [71,72,73]. Such synergy between cyanobacteria and *Vibrio* should be monitored as an element of bathing water quality assessment [74].

Along with the ecological significance, the investigations into the diversity, biological activities of natural products and their specific biotechnological applications were important elements of the study. In the assays, the inhibition of antibiotic-resistant *E. faecium* 45, isolated from the Sewage Treatment Plant, was observed (Table 2). The extracts were more active against *E. faecium* 45 than the tested fractions. It is believed that in some cases the mixtures of bioactive secondary metabolites could act more efficiently, compared with separated (or pure) metabolites. Such effects can be attributed to additive or synergistic interactions between many different compounds [75]. Moreover, the sample processing may lead to the loss of activity as a consequence of compound degradation or modification. The nutrients removal efficiencies in wastewater treatment plants based on cyanobacteria or microalgae species are well documented [76], while their role and efficiency in pathogen removal are still under investigation [77]. The wastewater treatment systems do not entirely eliminate antibiotic-resistant strains of enterococci in the treated water [77,78], therefore phytoplankton species, or especially cyanobacteria, might assist in reducing pathogens and fecal bacteria present in wastewaters.

Pharmacy is another important field where the application of natural products has great potential [79,80]. Extracts and fractions tested during our study revealed antibacterial activity against clinical, antibiotic-resistant bacterial strains (Table 2 and Table 4). Antibacterial activity of eukaryotic microalgae and cyanobacteria extracts, containing different classes of metabolites, is quite often reported in other studies [17,42,81]. During our study, the fractions eluted from solid-phase extraction cartridge with the more hydrophobic solvent (70–100% methanol in MiliQ water) were active against antibiotic-resistant Gram-positive bacteria. The mechanisms of action of cyanobacterial and microalgal metabolites against bacterial cells are not well described [82]. It has been proposed that the resistance of Gram-negative bacteria to metabolites produced by cyanobacteria is due to a hydrophilic outer membrane that blocks the penetration of hydrophobic metabolites through the cell membrane [83].

Our results also revealed high inhibitory activity of the tested extracts and fractions against serine proteases, trypsin, chymotrypsin and thrombin (Table 3 and Table 4); activity against T47D cells also confirmed pharmaceutical potential of phytoplankton natural products. Serine proteases have different roles related to human health (digestion, immune response or blood coagulation) [84]. Cyanobacterial secondary metabolites, such as CPs and AERs are generally considered to be the main classes of cyanopeptides responsible for the inhibition of serine proteases [26,58,85,86]. CPs and related depsipeptides also have cytotoxic activities [87]. Amongst CPs, microcystilide A showed cell-differentiation-promoting activity using HL-60 human leucocytes [88]. Research done by Salem et al. [89], showed that *Microcystis* extracts containing CPs, MGs and other metabolites were active against HepG-2, colon CaCO-2 and breast MCF-7 cancer cell lines. Additionally, MGs congeners showed the cytotoxic effect. The compounds were active towards human hepatocellular carcinoma (HepG2) cell line and had genotoxic activity [90]. MGs are also known to be angiotensin-converting enzyme (ACE) inhibitors [91], leucine aminopeptidase, aminopeptidase M, bovine aminopeptidase N and trypsin inhibitors [92,93]. Metabolites produced by other organisms present in bloom samples were also reported to have interesting biological activities. For example, the extracts of *Nitzschia* (diatom) cells exhibited ACE-inhibitory activity [94]; sulfolipids, isolated from several species of *Scenedesmus* (green microalgae) were effective in inhibiting alpha-glucosidase, glutaminyl-peptide cyclotransferase and telomerase [95]. Diatoms are considered to be a source of promising anticancer agents, such as *Synedra acus* produce chrysolaminaran, which exhibits HT-29 and DLD-1 colon cancer cells [96]; the extracts of *Melosira* and *Nitzschia* induce IPC-81 leukemia cell death [97].

Our results support the idea that the biodiscovery of new compounds in the environment that have dynamic conditions (lagoons, estuaries) provides a relatively high diversity of bioactive metabolites with biotechnological potential [28]. The promising results obtained in this work, encourage us for further studies into the structure, activity and application of specific metabolites produced by microorganisms isolated from Curonian Lagoon.

## Figures and Tables

**Table 1:** Testing steps and assays performed with extracts and fractions of the collected samples (N represents Nida site, J—Juodkrante site).

Collected Samples		1st Testing Step		2nd Testing Step			3rd Testing Step		
Sampling dates	Sample ID	Extract ID	Assay	ID ^1^ of the fractions tested		Assay	ID ^2^ of the fractions tested		Assay
N2018.06.24	1	I	acute toxicity, antibacterial, enzyme inhibition, cytotoxicity	I fractionation	−	−	II fractionation	−	−
N2018.06.28	2	II			−	−		−	−
J2018.07.11	3	III			−	−		−	−
J2018.07.20	4	IV			IV-[10–100] ^3^	antibacterial, cytotoxicity		IV-60-[20; 40; 60; 90; 100] ^3^; IV-70-[20; 40; 60; 90; 100] ^3^	cytotoxicity
N2018.07.23	5	V			V-[10–100] ^3^	antibacterial		−	−
N2018.08.03	6	VI			−	−		−	−
N2018.08.09	7	VII			−	−		−	−
N2018.08.16	8	VIII			VIII-[10–100] ^3^	enzyme inhibition		−	−
N2018.08.30	9	IX			IX-[10–100] ^3^			−	−

^1^—sample ID indicates: extract number (I–IX); I fractionation, fraction number; ^2^—sample ID indicates: extract number; I fractionation, fraction number; II fractionation, fraction number; ^3^ —numbers in square brackets represent eluent from 10% to 100% methanol (for 2nd Testing Step—every 10% (10 fractions for each extract); for 3rd Testing Step—20%, 40%, 60%, 90% and 100% methanol in MilliQ water); “−”—not tested.

**Table 2:** Antibacterial activity of extracts obtained from the Curonian Lagoon phytoplankton. Results are expressed as a percentage of bacterial culture OD value compared to untreated control (100% growth). Different colors highlight the differences in OD values of bacterial cultures (the color code is explained below the table).

Bacterial Strains		Clinical Strains												Environmental Strains											
		*Staphylococcus aureus* CCNPB/1505			*Pseudomonas aeruginosa* CCNPB/MBL			*Acinetobacter baumanii* CCNPB/O			*Klebsiella pneumoniae* CCNPB/1404			*Enterococcus faecium* 45			*Aeromonas salmonicida* 2013			*Vibrio cholerae* 2329			*Vibrio diazotrophicus* Cd1		
**Conc.,** **µg mL^−1^**		500	250	125	500	250	125	500	250	125	500	250	125	500	250	125	500	250	125	500	250	125	500	250	125
**Extracts**	I																								
	II																								
	III																								
	IV																								
	V																								
	VI																								
	VII																								
	VIII																								
	IX																								
**0–20%**		20–50%			50–70%			70–100%			100–120%			120–150%											

**Table 3:** Enzyme inhibition, cytotoxicity activity, and acute toxicity of phytoplankton extracts. Results are expressed as a percentage value of enzyme inhibition, cell viability (cytotoxicity assay) and cladocerans viability (acute toxicity assay), compared to untreated control. Different colors highlight the differences in values (the color code is explained below the table).

Bioassays		Enzyme Inhibition Assay				Cytotoxicity Assay				Acute Toxicity Assay					
		Trypsin		Thrombin		T47D Cells				*Daphnia Magna*					
**Conc.,** **µg mL^−1^**		45	4.5	45	4.5	200	100	50	25	24 h			48 h		
										10	5	2.5	10	5	2.5
**Extracts**	I														
	II														
	III														
	IV														
	V														
	VI														
	VII														
	VIII														
	IX														
High enzymatic inhibition/ Low cell viability/High toxicity ^1^				Low enzymatic inhibition/ High cell viability/Low toxicity ^1^											
0–20%		20–50%		50–70%		70–100%			100–120%						

^1^-percentage of live cladocerans.

**Table 4:** Antibacterial and cytotoxicity activities, and enzyme inhibition of fractions obtained after further separation of the extracts IV, V, VIII and IX.

Antibacterial Assay																				
Extracts	IV										V									
**Fractions ^1^**	10	20	30	40	50	60	70	80	90	100	10	20	30	40	50	60	70	80	90	100
*Staphylococcus aureus* CCNPB/1505	−		−	−	−	−	+	++	+	+	−	−	−	−	−	−	+	+	++	+
*Pseudomonas aeuruginosa* CCNPB/MBL	−		−	−	−	−	−	−	−	−	−	−	−	−	−	−	−	−	−	−
*Enterococcus faecium* 45	−		−	−	−	−	−	−	−	−	−	−	−	−	−	−	−	−	−	−
*Vibrio cholerae* 2329	−		−	−	−	−	++	++	−	++	−	−	−	−	−	−	−	+	++	+
*Aeromonas salmonicida* 2013	−		+	+	+	++	++	++	++	+	+	+	+	++	+	+	+	+	+	++
*Vibrio diazotrophicus* Cd1	+		+	+	++	+	+	−	+	+	+	++	+	+	+	+	+	+	+	+
**Cytotoxicity Assay**																				
**Extracts**	**IV**																			
**Fractions ^1^**	10	20	30	40	50	60	70	80	90	100										
T47D	−		−	−	−	++	++	−	−	−										
Fractions (3rd testing step)	**IV-50**					**IV-60**					**IV-70**									
	20	40	60	90	100	20	40	60	90	100	20	40	60	90	100					
T47D	−	−	−	−	−	−	−	−	−	−	−	−	−	−	−					
**Enzyme Inhibition Assay**																				
**Extracts**	**VIII**										**IX**									
**Fractions ^1^**	10	20	30	40	50	60	70	80	90	100	10	20	30	40	50	60	70	80	90	100
Trypsin	−	−	−	+	+	+	++	++	+	+	−	−	+	++	+	+	+	++	+	+
Chymotrypsin	−	−	−	−	+	+	++	+	+	+	−	−	−	+	+	+	+	+	+	+
Thrombin	−	−	−	+	+	+	+	+	+	−	−	−	−	+	++	+	+	+	−	−

^1^—names of fractions (10–100; concentration of MeOH in water (10–100%) used to elute the fractions); “++” indicates bacterial growth/enzymatic/cell viability inhibition by more than 80%; “+” inhibition in the range 50–80%; “−” inhibition lower than 50%. Markings have been applied based on the activity obtained from the lowest tested concentration.

## Data Availability

Not applicable.

## Supplementary Materials

The following are available online at https://www.mdpi.com/article/10.3390/biom11081139/s1, Figure S1: Phytoplankton abundance and biomass analysis, Figure S1: nMDS plot based on presence/absence Jaccard similarity matrix of Bacillariophyta (a), Chlorophyta (b) and Cyanophyta (c) communities, Figure S2: The total ion current (TIC) spectra of fractions from phytoplankton samples: IV-70 (a), IV-80 (b), V-70 (c), V-90 (d), IV-70-90 (e), VIII-70 (f), VIII-80 (g), IX-40 (h), IX-50 (i), IX-80 (j), Figure S3: The enhanced product ion mass spectrum of cyanopeptoline CP1055 with the suggested structure OA + Gln − [Thr^1^ + Tyr^2^ + Ahp^3^ + Phe^4^ + MeTyr^5^ + Val^6^] and the following fragment ions: *m*/*z* 939 [M + H − H_2_O − Val]^+^, 861 [M + H − H_2_O − MeTyr]^+^, 783 [M + H − H_2_O − (OA + Gln)]^+^, 765 [M + H − 2H_2_O − (OA + Gln)]^+^, 666 [M + H − 2H_2_O − (OA + Gln) − Val]^+^, 420 [Ahp + Phe + MeTyr + H − H_2_O]^+^, 150 MeTyr immonium; 136 Tyr immonium; 120 Phe immonium (OA − octanoic acid), Figure S4: The enhanced product ion mass spectrum of cyanopeptoline CP1012 with the suggested structure Ac + Gln − [Thr^1^ + Arg^2^ + Ahp^3^ + Phe^4^ + ClMeTyr^5^ + Ile^6^] and the following fragment ions: *m*/*z* 1013 [M + H]^+^, 995 [M + H − H_2_O]^+^, 967 [M + H − H_2_O − CO]^+^, 882 [M + H − H_2_O − Ile]^+^, 864 [M + H − 2H_2_O − Ile]^+^, 842 [M + H − H_2_O − (Ac + Gln)]^+^, 824 [M + H − 2H_2_O − (Ac + Gln)]^+^, 796 [M + H − 2H_2_O − (Ac + Gln) − CO]^+^, 455 [Ahp + Phe + ClMeTyr + H − H_2_O]^+^, 428 [Ac + Gln + Thr + Arg + H]^+^, 184 ClMeTyr immonium; 120 Phe immonium (Ac − acetyl group), Figure S5: The enhanced product ion mass spectrum of cyanopeptoline CP986 with the suggested structure HA + Gln − [Thr^1^ + Arg^2^ + Ahp^3^ + Leu^4^ + MeTyr^5^ + Val^6^] and the following fragment ions: *m*/*z* 987 [M + H]^+^, 969 [M + H − H_2_O]^+^, 941 [M + H − H_2_O − CO]^+^, 870 [M + H − HA − H_2_O]^+^, 760 [M + H − (HA + Gln)]^+^, 742 [M + H − (HA + Gln) − H_2_O]^+^, 714 [M + H − (HA + Gln) − H_2_O − CO]^+^, 584 [M + H − (Ahp + Leu + MeTyr)]^+^, 484 [HA + Gln + Thr + Arg + H]^+^, 386 [Ahp + Ile + MeTyr + H − H_2_O]^+^, 209 [Ahp + Ile + H − H_2_O]^+^, 181 [Ahp + Ile + H − H_2_O − CO]^+^, 150 MeTyr immonium, 136 Tyr immonium, 86 Ile immonium (HA − hexanoic acid), Figure S6: The enhanced product ion mass spectrum of microcystin [Ser^1^]MC − HTyrR with the structure Adda − [Glu + Mdha + Ser + HTyr + MeAsp + Arg] and the following fragment ions: *m*/*z* 941 [M + H − Adda]^+^, 923 [C_11_H_14_O + Glu + Mdha + Ser + HTyr + MeAsp + Arg + H]^+^, 918 [Adda + Glu + Mdha + Ser + HTyr + MeAsp + H]^+^, 863 [Ser + HTyr + MeAsp + Arg + Adda + H]^+^, 728 [MeAsp + Arg + Adda + Glu + H]^+^, 682 [Arg + Adda + Glu + Mdha + H]^+^, 633 [Mdha + Ser + HTyr + MeAsp + Arg + H]^+^, 599 [Arg + Adda + Glu + H]^+^, 550 [Ser + HTyr + MeAsp + Arg + H]^+^, 470 [Arg + Adda + H]^+^, 375 [C_11_H_14_O + Glu + Mdha + H]^+^, 213 [Glu + Mdha + H]^+^, 135 Adda fragment, Figure S7: The enhanced product ion mass spectrum of microginin MG928 with the suggested structure MeAhda + Phe + MeLeu + HTyr + Pro + Tyr and the following fragment ions: *m*/*z* 929 [M + H]^+^, 748 [M + H − Tyr]^+^, 651 [M + H − (Tyr + Pro)]^+^, 633 [M + H − (Pro + Tyr) − H_2_O]^+^, 474 [M + H − (HTyr + Pro + Tyr)]^+^, 456 [M + H − (HTyr + Pro + Tyr) − H_2_O]^+^, 348 [MeAhda + Phe + H]^+^, 330 [MeAhda + Phe + H − H_2_O]^+^, 182 [MeAhda − H_2_O]^+^, 172 [MeAhda − CO]^+^, 150 Hty immonium ion, 142 MeAhda fragment, 120 Phe immonium, 100 MeLeu immonium, Figure S8: The enhanced product ion mass spectrum of microginin MG783 with the suggested structure MeAhda + Val + MeLeu + HTyr + Tyr and the following fragment ions: *m*/*z* 784 [M + H]^+^, 603 [M + H − Tyr]^+^, 426 [M + H − (HTyr + Tyr)]^+^, 408 [M + H − (HTyr + Tyr) − H_2_O]^+^, 299 [M + H − (MeLeu + HTyr + Tyr)]^+^, 281 [M + H − (MeLeu + Htyr + Tyr) − H_2_O]^+^, 182 [MeAhda − H_2_O]^+^, 172 [MeAhda − CO]^+^, 142 MeAhda fragment, 100 MeLeu, Figure S9: The enhanced product ion mass spectrum of anabaenopeptin AP885CL with the suggested structure MeHTyr + CO − [Lys + Ile + Hty + MeAla + Phe] and the following fragment ions: *m*/*z* 886 [M + H]^+^, 868 [M + H − H_2_O]^+^, 858 [M + H − CO]^+^, 773 [M + H − Ile]^+^, 709 [M + H − HTyr]^+^, 691 [M + H − HTyr − H_2_O]^+^, 651 [M + H − (CO + MeHTyr)]^+^, 633 [M + H − (CO + MeHTyr) − H_2_O]^+^, 405 [M + H − MeHTyr − (Hty + Ile)]^+^, 320 [M + H − MeHTyr − (MeAla + Hty + Ile)]^+^, 263 [MeAla + Hty + H]^+^, 120 Phe immonium, 107 Tyr/HTyr, 84 Lys, Figure S10: The enhanced product ion mass spectrum of anabaenopeptin AP841CL with the suggested structure Tyr + CO − [Lys + Ile + Hph + MeAla + Phe] and the following fragment ions: *m*/*z* 842 [M + H]^+^, 824 [M + H − H_2_O]^+^, 814 [M + H − CO]^+^, 729 [M + H − Ile]^+^, 681 [M + H − Hph]^+^, 661 [M + H − Tyr − H_2_O]^+^, 635 [M + H − (CO + Tyr)]^+^, 405 [M + H –Tyr − (Hph + Ile)]^+^, 387 [M + H − Tyr − (Hph + Ile) − H_2_O]^+^, 320 [M + H − Tyr − (MeAla + Hph + Ile)]^+^, 247 [MeAla + Hph + H]^+^, 120 Phe immonium, 84 Lys, Figure S11: The enhanced product ion mass spectrum of anabaenopeptin AP915 with the suggested structure Tyr + CO − [Lys + Val + HTyr + MeHTyr + Ile] and the following fragment ions: *m*/*z* 916 [M + H]^+^, 898 [M + H − H_2_O]^+^, 888 [M + H − CO]^+^, 817 [M + H − Val]^+^, 803 [M + H − Ile]^+^, 739 [M + H − HTyr]^+^, 721 [M + H − HTyr − H_2_O]^+^, 725 [M + H − MeHTyr]^+^, 709 [M + H − (CO + Tyr)]^+^, 612 [M + H − (MeHTyr + Ile)]^+^, 477 [M + H − Tyr − (HTyr + Val)]^+^, 369 [MeHTyr + HTyr + H]^+^, 305 [MeHTyr + Ile + H]^+^, 164 MeHTyr immonium, 84 Lys, Figure S12: The enhanced product ion mass spectrum of aeruginosin AER618 with the suggested structure Hpla + Leu/Ile + Choi + Arg and the following fragment ions: *m*/*z* 619 [M + H]^+^, 601 [M + H − H_2_O]^+^, 559 [M + H − H_2_O − CH_2_N_2_]^+^, 455 [M + H − Hpla]^+^, 325 [Choi + Arg + H − NH_3_]^+^, 307 [Choi + Arg + H − NH_3_ − H_2_O]^+^, 282 [Choi + Arg + H − CH_2_N_2_ − H_2_O]^+^, 70 and 175 Arg, 122 and 140 Choi ions, 86 Leu/Ile immonium, Table S1: Bacterial strains used in the antibacterial activity assay, Table S2: Microcystins and other oligopeptides detected in the fractions obtained from the Curonian Lagoon. Red color indicates active fractions; X indicates non-determined amino acids; empty cells—microcystins and other oligopeptides not detected, Table S3: Antibacterial activity of extracts obtained from Curonian Lagoon phytoplankton. Results are expressed as a percentage of bacterial culture OD value compared to untreated control (100% growth). Different colors highlight the differences in OD values of bacterial cultures (the color code is explained below the table), Table S4: Enzyme inhibition, cytotoxicity activity, and acute toxicity of phytoplankton extracts. Results are expressed as a percentage value of enzyme inhibition, cell viability (cytotoxicity assay) and cladocerans viability (acute toxicity assay), compared to untreated control. Different colors highlight the differences in values (the color code is explained below the table), Table S5: Antibacterial activity of fractions obtained after further separation of the extracts IV and V. Results are expressed as a percentage of bacterial culture OD value compared to untreated control (100% growth). Different colors highlight the differences in OD values of bacterial cultures (the color code is explained below the table), Table S6: Enzyme inhibition of fractions obtained after further separation of the extracts VIII and IX. Results are expressed as a percentage value of enzyme inhibition compared to untreated control. Different colors highlight the differences in values (the color code is explained below the table), Table S7: T47D cancer cells viability of fractions obtained after further separation of the extracts IV, and fractions IV-50, IV-60 and IV-70. Results are expressed as a range of percentage effect compared to untreated control. Different colors have been applied to highlight the differences in values (the color code is explained below the table), Table S8: Presence and dominance of phytoplankton species in the samples (1–9) from the Curonian lagoon. Cyanobacteria dominance was expressed as a percentage of total cyanobacteria biomass. The dominance of other species of phytoplankton was expressed as a percentage of total phytoplankton biomass. The color code is explained below the table. Table S9. Microcystins (MCs) and other oligopeptides detected in the most active fractions obtained from the phytoplankton samples collected in the Curonian Lagoon (the numbers in parentheses are given to distinguish compounds with the same molar mass but different amino acid sequences).
