# Supplementary material for: Phytoplankton of the Curonian Lagoon as a New Interesting Source for Bioactive Natural Products. Special Impact on Cyanobacterial Metabolites
Source: Biomolecules. 2021 Aug 2;11(8):1139. doi: 10.3390/biom11081139 (PMC8395022; doi:10.3390/biom11081139)
Supplement: Supplementary file 1 [file biomolecules-11-01139-s001.zip › biomolecules-1285557-supplementary.pdf]

Supplementary Material

Phytoplankton abundance and biomass analysis.

According to HELCOM recommendations [30], the phytoplankton abundance (counts L^{-1}) was calculated by multiplying the number of units counted (filamentous cyanobacteria were counted in lengths of 100 μm as one count) with the coefficient C (dm^3), calculated using the following equation:

$C = (A \times 1000)/(N \times a \times V)$,

Where A is the cross-section area of the top cylinder of the combined sedimentation chamber (the usual inner diameter is 25.0 mm, giving A= 491 mm^2), N is the number of counted fields or transects, a is the area of a single field or transect, and V is the volume (cm^3) of sedimented aliquot.

The phytoplankton biomass (mg L^{-1}) was calculated by the allocation of phytoplankton species (genus) to size classes according to the scheme of Olenina et al. [31] and updated appendix available at ICES website (http://ices.dk/data/data-portals/Pages/DOME.aspx). The phytoplankton biomass (mg L^{-1}) was calculated based on the following equation, as recommended by HELCOM [30]:

$Biomass = abundance \times VCU \times 10^{-6}$,

Where VCU is the volume of the counting unit (μg).

Table S1. Bacterial strains used in the antibacterial activity assay.

Strains	Isolate's no.	Bacterial species	Gram stain	Collection code	Resistance pattern	Isolation source
Clinical	1	*Staphylococcus aureus*	+	CCNPB/1505	MRSA[4]	Cervix
	2	*Acinetobacter baumannii*	-	CCNPB/O	ESBL[5]	Urine
	3	*Klebsiella pneumoniae*	-	CCNPB/1404	ESBL	Urine
	4	*Pseudomonas aeruginosa*	-	CCNPB/MBL	MBL[6]	Wound
Environmental	5	*Aeromonas salmonicida*	-	2013[1]	not analyzed	Baltic Sea
	6	*Vibrio diazotrophicus*	-	Cd1	not analyzed	
	7	*Vibrio cholerae*	-	2329[2]	tetracycline	
	8	*Enterococcus faecium*	+	45[3]	Erythromycin, chloramphenicol, linezolid, nitrofurantoin	Aeration chamber, Gdansk-Wschód, Sewage Treatment Plant, Poland

[1] – [38]; [2] – [36]; [3] – [37]; [4]MRSA – methicillin-resistant *Staphylococcus aureus*; [5]ESBL – extended-spectrum β-lactamase; [6]MBL – metallo-β-Lactamase.

Table S2. Microcystins and other oligopeptides detected in the fractions obtained from the Curonian Lagoon. The red color indicates active fractions; X indicates non-determined amino acids; empty cells – microcystins and other oligopeptides not detected.

Detected metabolites	*m/z*	Structure	Antibacterial		Enzyme inhibition		Cytotoxicity			References
			Fractions							
			IV	V	VIII	IX	IV-50	IV-60	IV-70	
**AERUGINOSINS**										
AER552	553 (585)	115-Leu-Choi-Argal			VIII-70; VIII-80					This study
AER526	526	X-Leu-Choi-X							IV-70-60; IV-70-90	This study
AER567	568 (600)	115-Lys?-Choi-Argal			VIII-50; VIII-70; VIII-80; VIII-90; VIII-100	IX-50; IX-60; IX-70; IX-80; IX-90; IX-100				This study
AER579	580	X-Leu-Choi-Argal	IV-50				IV-50-20			This study
AER588	589 (607/625)	Hpla-Val-Choi-Argal			VIII-50; VIII-60; VIII-70; VIII-80; VIII-90	IX-60; IX-70				This study
K139	604 (621/635)	Hpla-Ile-Choi-Argal	IV-40; IV-60; IV-70	V-60; V-70	VIII-40; VIII-60; VIII-70	IX-70; IX-80	IV-50-20; IV-50-60	IV-60-20; IV-60-40; IV-60-60; IV-60-90	IV-70-20; IV-70-40; IV-70-60; IV-70-90	[57]
AER617	618	Hpla-Lys?-Choi-Argal			VIII-40-100	IX-40-60; IX-80; IX-100				This study
AER618	619	Hpla-Ile/Leu-Choi-Arg			VIII-70; VIII-80; VIII-90; VIII-100				IV-70-20	This study

AER636	637	ClHpla-Leu-Choi-Argal	IV-40; IV-70	V-40	VIII-50; VIII-70; VIII-80		IV-50-20		IV-70-60; IV-70-90	This study
AER638	639 (657)	X-X-Choi-Argal	IV-40; IV-50		VIII-50; VIII-60; VIII-70; VIII-80	IX-70				This study
AER640	641	Hpla-151-Choi-Argal			VIII-40					This study
AER652	653	Hpla-Tyr-Choi-Argal	IV-30; IV-50	V-30	VIII-40		IV-50-20; IV-50-40; IV-50-60			This study
AER682	683	SuHpla-Leu-Choi-Argal	IV-40							This study
**CYANOPEPTOLINS**										
CP1048	1049	X-X-[Thr-Arg-Ahp-Phe-MeTyr-Val]	-						IV-70-90	[86]
CP1045	1046	X-X-[Thr-Ile-Ahp-Phe-MeTyr-Ile]		V-70	VIII-70				IV-70-40; IV-70-60	This study
CP1055	1038	OA-Gln-[Thr-Tyr-Ahp-Phe-MeTyr-Val]	IV-70		VIII-80				IV-70-60	This study
CP1029	1030	Hpla-Asp-[Thr-Lys-Ahp-Phe-MeTyr-Val?]			VIII-80	IX-80				This study
CP1023	1024	X-X-[Thr-X-Ahp-Phe-MeTyr-X]	IV-60	V-70		IX-80		IV-60-60; IV-60-90		This study
CP?	1023	X-X-[Thr-Tyr-Ahp-X-MeTyr-X]						IV-60-20; IV-60-60		This study
CP1020	1021	OA-Gln?-[Thr-Lys-Ahp-Phe-MeTyr-Ile?]			VIII-90	IX-90				This study
CP1015	1016	X-X-[Thr-83(dhb?)-Ahp-Phe-MeTyr-Ile]	IV-70	V-70	VIII-80	IX-70; IX-80			IV-70-60; IV-70-90	This study
CP1014	1015	HA-Asp-[Thr-Arg-Ahp-Leu-MeTyr-Val]	IV-70	V-90					IV-70-60; IV-70-90	[26]

CP1012	1013	Ac-Gln-[Thr-Arg-Ahp-Phe-ClMeTyr-Ile]	IV-50	V-60; V-70; V-80	VIII-70; VIII-80; VIII-90	IX-70		IV-60-20; IV-60-90		[45,91]
CP1011	1011	X-X-[Thr-X-Ahp-Phe-MrTrp-MeTrp-X]			VIII-60		IV-50-20	IV-60-20; IV-60-40		This study
CP1009 (1037)	1009 (1039)	HA-Gln[Thr-Tyr-Ahp-Phe-MeTyr-Val]				IX-70				This study
CP?	1007 (877?)	To be determied						IV-60-40		This study
CP1006 (1)	1007	HA-Asp-[Thr-Arg-Ahp-Phe-MeTyr-Val]	IV-70	V-70; V-80	VIII-80; VIII-90	IX-80		IV-60-90	IV-70-20; IV-70-40; IV-70-60; IV-70-90	[46]
CP1006 (2)	1007	X-X-[Thr-Lys-Ahp-Phe-MeTyr-Val]	IV-80	V-80	VIII-90	IX-90			IV-70-90	This study
CP995	996	HA-Asp-[Thr-Tyr-Ahp-Phe-MeTyr-Val]	IV-50	V-60	VIII-60; VIII-70	IX-60	IV-50-20; IV-50-40; IV-50-60; IV-50-90	IV-60-40; IV-60-60		[86]
CP993	994	OA?-Ala (or HA-Asp)-[Thr-Ile-Ahp-Phe-MeTyrCl-Ile]	IV-70	V-60; V-70		IX-70	IV-50-40	IV-60-60	IV-70-20; IV-70-40; IV-70-60	This study
CP992	993	X-X-[Thr-Ile-Ahp-Phe-MeTyr-Ile]		V-80	VIII-80; VIII-90	IX-80			IV-70-60; IV-70-90	This study
CP989	990	X-X-[Thr-Tyr-Ahp-Phe-MeTyr-Val]	IV-60; IV-70	V-70	VIII-80	IX-80		IV-60-20; IV-60-40; IV-60-60; IV-60-90	IV-70-60	[86]
CP986 (1)	987	X-X-[Thr-X-Ahp-Phe-MeTyrCl-X]						IV-60-20		This study
CP986 (2)	987	HA-Gln-[Thr-Arg-Ahp-Leu-MeTyr-Val]	IV-70; IV-100	V-80; V-90	VIII-90	IX-100			IV-70-90	[46,47]
CP984	985	X-X-[Thr-X-Ahp-Phe-MeTyrCl-X]			VIII-60; VIII-70					This study

CP978 (1)	979	BA-Asp-[Thr-Arg-Ahp-Phe-MeTyr-Val]	IV-70	V-80	VIII-60; VIII-90	IX-80; IX-90			IV-70-60; IV-70-90	[45,86]
CP978 (2)	979	X-X-[Thr-X-Ahp-Phe-MeTyr-X]	IV-50; IV-60	V-60	VIII-60; VIII-70; VIII-80	IX-60	IV-50-20; IV-50-40; IV-50-60	IV-60-20	IV-70-20	This study
CP972	973	HA-Gln-[Thr-Lys-Ahp-Leu-MeTyr-Ile]	IV-70	V-90	VIII-100	IX-90			IV-70-60; IV-70-90	This study
CP970	971	X-X-[Thr-X-Ahp-Phe-MeTyr-X]	IV-60	V-60	VIII-50; VIII-60					This study
Micropeptin A	970 (987)	OA-Glu-[Thr-Lys-Ahp-Leu-MeTyr-Val]		V-90						[47,91]
CP986 (3)	967	BA-Asp-[Thr-Tyr-Ahp-Phe-MeTyr-Val]	IV-50; IV-60	V-60	VIII-50; VIII-60; VIII-70		IV-50-60	IV-60-20; IV-60-40; IV-60-60; IV-60-90	IV-70-20	[26,47,86]
CP965	966 (=986)	Ac-Gln-[Thr-Gln-Ahp-Phe-MeTyrCl-Ile]	IV-40; IV-50; IV-60	V-60		IX-60; IX-70	IV-50-20; IV-50-60	IV-60-20; IV-60-40; IV-60-60; IV-60-90	IV-70-20; IV-70-40	This study
CP979	962	HA-Asp-[Thr-Tyr-Ahp-Ile-MeTyr-Val]		V-60			IV-50-20; IV-50-40; IV-50-60; IV-50-90	IV-60-20; IV-60-60		This study
CP960 (978)	961 (979)	HA-Asp-[Thr-Lys-Ahp-Phe-MeTyr-Val]	IV-70	V-80	VIII-80; VIII-90				IV-70-60; IV-70-90	This study
Micropeptin B	959	HA-Glu-[Thr-Lys-Ahp-Leu-MeTyr-Val] or BA-Gln-[Thr-Arg-Ahp-Leu-MeTyr-Val]	IV-60	V-70; V-80	VIII-80; VIII-90	IX-80		IV-60-60; IV-60-90	IV-70-20; IV-70-60; IV-70-90	[26,91]
CP956	957	BA-Ala-Abu-[Thr-Leu-Ahp-Phe-MeTyr-Val]		V-60						This study
CP970	953	X-X-[Thr-Tyr-Ahp-Phe-MeTyr-Val]		V-60			IV-50-20; IV-50-40; IV-50-60	IV-60-20; IV-60-40; IV-60-60		This study

CP951	952	X-X-[Thr-Ile-Ahp-Phe-MeTyrCl-Val]	IV-50; IV-60		VIII-60; VIII-70		IV-50-20; IV-50-60	IV-60-60		This study
CP946	947	X-X-[Thr-X-Ahp-Thr-MePhe-Ile]		V-70	VIII-60		IV-50-60		IV-70-60	This study
CP962	945/6	HA-Asp-[Thr-Tyr-Ahp-Ile-MeTyr-Val]	IV-70	V-50; V-70; V-80	VIII-60; VIII-80; VIII-90	IX-80		IV-60-60; IV-60-90	IV-70-20; IV-70-40; IV-70-60; IV-70-90	This study
CP939	940	X-X-[Thr-X-Ahp-Phe-MeTyr-X]	IV-50;	V-60	VIII-60		IV-50-20; IV-50-40; IV-50-60	IV-60-20		This study
CP936	937	X-X-[Thr-X-Ahp-Phe-MeTyr-X]	IV-50;	V-60	VIII-50; VIII-60; VIII-70		IV-50-20	IV-60-20		This study
CP930 (1)	931	X-X-[Thr-X-Ahp-Phe-MeTyr-Ile]			VIII-60			IV-60-60		This study
CP930 (2)	931	X-X-[Thr-X-Ahp-Phe-MeTyr-X]						IV-60-60		This study
CP926	927	HA-Asp-[Thr-Lys?-Ahp-Ile-MeTyr-Val]							IV-70-60	This study
CP937	920	X-X-[Thr-Tyr-Ahp-Ile-Metyr-X]					IV-50-20			This study
CP936	919	BA?-Gln?[Thr-Tyr-Ahp-Phe-MeTyr-X]			VIII-60				IV-70-60	This study
CP916 (CP931)	917 (932)	X-X-[Thr-X-Ahp-Phe-MeTyr-X]	IV-40; IV-50; IV-60; IV-70	V-50; V-60; V-70	VIII-60; VIII-70; VIII-80	IX-70	IV-50-20; IV-50-40; IV-50-60	IV-60-20; IV-60-40; IV-60-60; IV-60-90	IV-70-20; IV-70-90	This study
CP912	911	X-X-[Thr-Pro?-Ahp-Phe-MeTyrCl-Ile]	IV-40	V-40	VIII-40; VIII-50	IX-40	IV-50-60		IV-70-20	This study
CP902	903	X-X-[Thr-X-Ahp-Phe-MeTyr-Val]	IV-60; IV-70	V-60; V-70	VIII-60; VIII-70; VIII-80	IX-60; IX-70		IV-60-20; IV-60-40; IV-60-60	IV-70-20; IV-70-40; IV-70-60	This study
CP895	896	X-[Thr-X-Ahp-Phe-MeTyr-X]	IV-30; IV-40; IV-50	V-40; V-50; V-60	VIII-40; VIII-60	IX-60	IV-50-20; IV-50-40; IV-50-60		IV-70-20	This study

CP887	888	X-X-[Thr-X-Ahp-Phe-MeTyr-Ile]								This study
CP886 (1)	887	AA-Gln-[Thr-X-Ahp-Phe-MeTyrCl-Ile]			VIII-80	IX-60		IV-60-40; IV-60-60	IV-70-90	This study
CP886 (2)	887	X-X-[Thr-X-Ahp-Phe-MeTyr-Ile]	IV-50; IV-60	V-60	VIII-60; VIII-70	IX-60	IV-50-60			This study
CP887	878	X-X-[Thr-X-Aph-Ahp-Phe-MeTyr-X]		V-40; V-60	VIII-40; VIII-60		IV-50-20			This study
CP871	872	X-[Thr-X-Ahp-Phe-MeTrp-X]	IV-50	V-40; V-50	VIII-50	IX-40				This study
**MICROGININS**										
928	929	MeAhda+Phe+MeLeu+Htyr+Pro+Tyr	IV-70						IV-70-90	This study
783	784	MeAhda+Val+MeLeu+Htyr+Tyr	IV-70						IV-70-90	This study
769	770	MeAhda+Val+Leu+Hty+Tyr		V-70	VIII-80; VIII-90	IX-80		IV-60-90	IV-70-60; IV-70-90	[54]
KR767	768	MeAhda+Tyr+MeLeu+Pro+Tyr	IV-70	V-70	VIII-80; VIII-90	IX-80		IV-60-90	IV-70-60; IV-70-90	[53]
755	756	MeAhda+Ala+MeLeu+Hty+Tyr	IV-60; IV-70	V-70	VIII-80; VIII-90					[53]
753	754	Ahda+Tyr+MeIle/MeLeu+Pro+Tyr	IV-60; IV-70	V-70	VIII-80; VIII-90	IX-70; IX-80		IV-60-60; IV-60-90	IV-70-60; IV-70-90	This study
748?	749	To be determined	IV-70		VIII-80; VIII-90	IX-80				This study
744	744	Ahda-MeSer-Ile-Tyr-Tyr			VIII-70; VIII-80			IV-60-20; IV-60-40		[53]
743?	744	Cl_2MeAhda+Ala+Ile+Pro+Hty	IV-70		VIII-80; VIII-90			IV-60-60; IV-60-90	IV-70-60; IV-70-90	This study
741	742	Ahda+Hty+Val+Val+Tyr	IV-70	V-70	VIII-80; VIII-90	IX-70; IX-80			IV-70-60; IV-70-90	This study
FR1	729	Ahda+Ala+MeLeu+Tyr+Tyr	IV-70		VIII-80; VIII-90			IV-60-20	IV-70-60; IV-70-90	[53]

FR3	727	Ahda+Thr+Pro+Tyr+Tyr	IV-70	V-50; V-60; V-70	VIII-60; VIII-80; VIII-90	IX-60; IX-70; IX-80	IV-50-20; IV-50-60	IV-60-20; IV-60-40; IV-60-60; IV-60-90	IV-70-20; IV-70-60; IV-70-90	[26]
FR5	726	Ahda+Val+Pro+Tyr+Tyr	IV-50; IV-60	V-70	VIII-70; VIII-90	IX-70		IV-60-20; IV-60-40; IV-60-60; IV-60-90	IV-70-20; IV-70-60; IV-70-90	[26]
713	714	Ahda+Ala+Ala+Val+MeTyr+Tyr	IV-60; IV-70		VIII-60; VIII-70; VIII-80; VIII-90	IX-70		IV-60-20; IV-60-40; IV-60-60; IV-60-90	IV-70-20; IV-70-60; IV-70-90	[91]
678	679	MeAhda+Phe+MeLeu+Tyr	IV-70						IV-70-60; IV-70-90	This study
650+H2O	651	MeAhda+MeLeu+Phe+Hty			VIII-80				IV-70-90	This study
642	643	Ahoa+Tyr+MeAla+Tyr	IV-60	V-70						This study
636+H2O?	637	Mehda+Phe+MeIle+Tyr+H_2O?	IV-40	V-40			IV-50-20		IV-70-60	This study
**ANABAENOPEPTINS**										
[Hph^4] AP F	821	ArgCO-[Lys+Val+Hph+MeAla+Phe]	IV-60	V-70		IX-70	IV-50-20	IV-60-20; IV-60-40; IV-60-60; IV-60-90	IV-70-20; IV-70-60	[26]
AP D	828	[Phe-MeAla-HTyr-Val-Lys]-CO-Phe	IV-40; IV-50	V-50	VIII-50		IV-50-20			[56]
AP B	837	ArgCO-[Lys+Val+Hty+MeAla+Phe]	IV-50; IV-60	V-50; V-60	VIII-50; VIII-60	IX-60	IV-50-20; IV-50-40; IV-50-60	IV-60-20; IV-60-60		[55]
AP841CL	842	TyrCO[Lys+Ile+Hph+MeAla+Phe]	IV-50		VIII-60		IV-50-40; IV-50-60			This study
AP A	844	TyrCO-[Lys+Val+Hty+MeAla+Phe]	IV-30; IV-40	V-40	VIII-40; VIII-50	IX-40; IX-50				[55]

Unknown	848	IleCO-[Lys+Val+Hty+X+X]	IV-40; IV-60	V-60; V-70	VIII-60; VIII-70	IX-70		IV-60-20; IV-60-40; IV-60-60; IV-60-90	IV-70-60	This study
AP F	851	ArgCO-[Lys+Ile+Hty+MeAla+Phe]	IV-50; IV-60	V-50; V-60	VIII-60; VIII-70	IX-60	IV-50-20; IV-50-40; IV-50-60	IV-60-20; IV-60-40; IV-60-60	IV-70-20	[55]
Oscillamide Y	858	TyrCO-[Lys+Ile+Hty+MeAla+Phe]	IV-40; IV-50	V-40; V-50	VIII-50; VIII-60	IX-50	IV-50-20; IV-50-40; IV-50-60		IV-70-20	[56]
AP871CL	872	MeHTyrCO-[Lys+Val+Hty+MeAla+Phe]	IV-40; IV-50	V-50	VIII-50; VIII-60		IV-50-20; IV-50-40; IV-50-60			This study
AP873CL	874	TyrCO-[Lys+Ile+Hty+MeSer?+Phe]	IV-40	V-40						This study
AP884CL	885	ArgCO-[Lys+MeO+Hty+MeAla+MeO]	IV-50		VIII-50					This study
AP885CL	886	MeHTyrCO-[Lys+Ile+Hty+MeAla+Phe]	IV-50	V-50	VIII-50		IV-50-20; IV-50-40; IV-50-60			This study
AP 915	916	TyrCO-[Lys+Val+Htyr+MeHtyr+Ile]	IV-40; IV-50	V-50			IV-50-20; IV-50-40; IV-50-60			[56]
AP G	930	TyrCO-[Lys+Ile+Htyr+MeHtyr+Ile]			VIII-40; VIII-60		IV-50-60			[55]
**MICROCYSTINS**										
[Ser[1]]MC-HtyR	1075	Cyclo[Adds-Glu-Mdha-Ser-Htyr-MeAsp-Arg]	IV-40; IV-50	V-50	VIII-50		IV-50-20			[49,51,52]
MC1068	1068	Cyclo[Adda-Glu-Mdha-Ala-Trp-MeAsp-Arg]			VIII-60; VIII-80	IX-60				This study
MC-WR	1057	Cyclo[ADMAdda-Glu-Mdha-Ala-Phe-MeAsp-Arg]	IV-50	V-50	VIII-50					[49,51,52]
MC1049	1049	Cyclo[Adda-Glu-Mdha	IV-40	V-40; V-50	VIII-50		IV-50-20			This study

MC-YR	1045	Cyclo[Adda-Glu-Mdha-Ala-Tyr-MeAsp-Arg]	IV-40; IV-50	V-50	VIII-50	IX-50	IV-50-20; IV-50-60			[49,51,52]
MC-HphR	1043	Cyclo[Adda-Glu-Mdha-Ala-Hph-MeAsp-Arg]	IV-50	V-50; V-60	VIII-80	IX-80	IV-50-60			[48,49,51,52]
MC-RR	1038	Cyclo[Adda-Glu-Mdha-Ala-Arg-MeAsp-Arg]	IV-50; IV-60; IV-70	V-50; V-60; V-70; V-80	VIII-60; VIII-70; VIII-80; VIII-90; VIII-100	IX-60; IX-70; IX-80	IV-50-20; IV-50-60	IV-60-20; IV-60-40; IV-60-60; IV-60-90	IV-70-20; IV-70-40; IV-70-60; IV-70-90	[49,51,52]
[Asp^{3}Dha^7]MC-YR	1031	Cyclo[Adda-Glu-Mdha-Ala-Tyr-Asp-Arg]	IV-50		VIII-90		IV-50-20; IV-50-60			[49,51,52]
MC-FR	1029	Cyclo[Adda-Glu-Mdha-Ala-Phe-MeAsp-Arg]	IV-40; IV-50	V-40; V-50	VIII-50		IV-50-20; IV-50-60			[49,50,52]
MC-LW	1025	Cyclo[Adda-Glu-Mdha-Ala-Leu-MeAsp-Trp]			VIII-70; VIII-80					[49,51,52]
MC-HilR	1009	Cyclo[Adda-Glu-Mdha-Ala--MeAsp-Arg]	IV-50	V-50	VIII-60; VIII-80		IV-50-20; IV-50-60			[49,51,52]
MC-LY	1002	Cycylo[Adda-Glu-Mdha-Ala-Leu-MeAsp-Tyr]			VIII-60					[49,51,52]
MC-LR	995	Cyclo[Adda-Glu-Mdha-Ala-Leu-MeAsp-Arg]	IV-40; IV-50; IV-60	V-50	VIII-50; VIII-60; VIII-70; VIII-80	IX-50; IX-60	IV-50-20; IV-50-40; IV-50-60; IV-50-90			[49,51,52]
[Asp^3]MC-LY	988	Cyclo[Adda-Glu-Mdha-Ala-Leu-Asp-Tyr]			VIII-60					[49,51,52]
MC-LF	986	Cycylo[Adda-Glu-Mdha-Ala-Leu-MeAsp-Phe]	IV-70		VIII-40; VIII-50; VIII-80				IV-70-60	[49,51,52]
[Asp^3]MC-LR	981	Cycylo[Adda-Glu-Mdha-Ala-Leu-MeAsp-Arg]	IV-40; IV-50	V-50			IV-50-60	IV-60-20		[49,51,52]
[Dha^7]MC-LR	981	Cycylo[Adda-Glu-Dha-Ala-Leu-MeAsp-Arg]		V-60	VIII-60; VIII-80	IX-60	IV-50-20; IV-50-60			[49,51,52]
[Asp^{3}Dha^7]MC-RHty	1031	Cycylo[Adda-Glu-dha-Ala-Leu-MeAsp-HTyr]	IV-50; IV-70		VIII-90; VIII-100	IX-50; IX-90; IX-100	IV-50-20		IV-70-60	[49,51,52]

[Dha⁷]MC-RR	1024	Cycylo[Adda-Glu-Dha-Ala-Arg-MeAsp-Arg]	IV-50; IV-60	V-50; V-60; V-70	VIII-60; VIII-70; VIII-80; VIII-90	IX-70	IV-50-20	IV-60-20; IV-60-40; IV-60-60	IV-70-60	[49,51,52]

Table S3. Antibacterial activity of extracts obtained from the Curonian Lagoon phytoplankton. Results are expressed as a percentage of bacterial culture OD value compared to untreated control (100% growth). Different colors highlight the differences in OD values of bacterial cultures (the color code is explained below the table).

Clinical strains		*Staphylococcus aureus* CCNPB/1505			*Pseudomonas aeruginosa* CCNPB/MBL			*Acinetobacter baumanii* CCNPB/O			*Klebsiella pneumoniae* CCNPB/1404		
Conc., ug/mL		500	250	125	500	250	125	500	250	125	500	250	125
Extracts	I	72	82	80	75	85	96	93	89	9	96	90	89
	II	12	22	84	88	107	112	90	90	92	87	82	82
	III	49	91	90	102	105	114	87	86	95	94	87	85
	IV	11	13	66	57	90	110	9	89	88	84	84	80
	V	15	27	75	49	7	95	89	93	90	94	94	90
	VI	42	66	83	7	105	114	8	8	92	86	82	83
	VII	58	73	81	8	97	104	103	93	91	105	89	88
	VIII	75	76	81	61	60	72	96	88	90	8	83	81
	IX	60	54	78	73	101	82	101	91	84	92	80	83
Environmental strains		*Enterococcus faecium* 45			*Aeromonas salmonicida* 2013			*Vibrio cholerae* 2329			*Vibrio diazotrophicus* Cd1		
Conc., ug/mL		500	250	125	500	250	125	500	250	125	500	250	125
Extracts	I	35	62	96	20	30	28	68	94	104	15	19	75
	II	35	33	63	18	25	23	76	94	101	11	12	14
	III	35	68	82	19	20	26	52	88	101	11	14	19
	IV	29	29	32	2	55	71	61	103	95	31	56	63
	V	35	34	83	30	23	23	113	134	119	38	29	30
	VI	34	31	80	10	19	24	77	99	106	8	13	17
	VII	43	52	77	8	19	23	92	103	105	6	11	85
	VIII	67	76	79	95	105	100	103	100	106	79	67	91
	IX	49	56	73	77	105	93	95	87	105	94	106	94

0-20%	20-50%	50-70%	70-100%	100-120%	120-150%

Table S4. Enzyme inhibition, cytotoxicity activity, and acute toxicity of phytoplankton extracts. Results are expressed as a percentage value of enzyme inhibition, cell viability (cytotoxicity assay), and cladocerans viability (acute toxicity assay), compared to untreated control. Different colors highlight the differences in values (the color code is explained below the table).

Assays		Enzyme inhibition					Cytotoxicity					Acute toxicity					
		Trypsin		Thrombin			T47D cells					*Daphnia magna*					
												24 hours			48 hours		
Conc., µg/mL		45	4.5	45	4.5		200	100	50	25		10	5	2.5	10	5	2.5
Extracts	I	12	82	23	94		53	70	74	76		73	82	82	62	42	48
	II	22	60	30	91		6	9	66	74		91	98	98	89	98	98
	III	19	70	21	90		12	65	66	109		91	91	98	89	91	58
	IV	23	87	29	97		4	31	46	70		82	98	98	58	91	55
	V	14	88	28	80		8	50	67	89		98	98	98	98	98	98
	VI	22	73	28	93		6	60	67	95		91	98	91	55	91	91
	VII	22	59	36	95		4	59	71	72		82	98	98	58	98	89
	VIII	8	10	24	26		48	74	87	94		98	98	98	89	91	65
	IX	16	40	32	47		52	72	84	100		98	91	91	91	78	71

High enzymatic inhibition/ Low cell viability/High toxicity[1]				Low enzymatic inhibition/ High cell viability/Low toxicity[1]
0-20%	20-50%	50-70%	70-100%	100-120%

[1]-percentage of live cladocerans

Table S5. Antibacterial activity of fractions obtained after further separation of the extracts IV and V. Results are expressed as a percentage of bacterial culture OD value compared to untreated control (100% growth). Different colors highlight the differences in OD values of bacterial cultures (the color code is explained below the table).

Bacterial strains			Gram-positive bacterial strains						Gram-negative bacterial strains											
			Staphylococcus aureus CCNPB/1505			*Enterococcus faecium* 45			*Pseudomonas aeruginosa* CCNPB/MBL			*Aeromonas salmonicida* 2013			*Vibrio cholerae* 2329			*Vibrio diazotrophicus* Cd1		
Conc., ug/mL			1000	500	250	1000	500	250	1000	500	250	1000	500	250	1000	500	250	1000	500	250
Fractions	IV	10, 20	75	73	75	76	64	66	113	111	104	55	64	67	101	102	99	30	97	98
		30	124	121	123	93	88	100	114	116	116	38	51	55	108	107	103	24	75	93
		40	107	104	115	71	53	60	124	129	120	29	24	23	141	129	117	20	22	21
		50	87	83	88	63	52	73	119	123	127	25	21	26	109	126	129	31	19	19
		60	56	80	91	92	73	78	114	128	129	21	17	18	103	119	127	26	22	24
		70	31	40	55	126	113	121	121	128	126	19	18	49	6	90	103	38	45	49
		80	18	17	34	72	122	151	92	142	138	33	19	19	6	115	120	139	91	85
		90	20	35	51	88	91	122	184	155	132	40	27	19	108	127	117	52	34	24
		100	42	65	71	75	73	71	120	105	109	38	26	25	12	110	109	39	32	32
	V	10	69	85	64	104	75	98	143	138	138	34	46	31	113	121	133	29	29	25
		20	63	62	62	112	95	128	121	118	127	25	22	58	120	119	128	27	25	19
		30	71	74	75	101	83	91	115	118	125	22	21	48	122	134	120	32	34	86
		40	58	61	65	94	80	87	102	104	108	25	19	21	117	121	134	26	25	27
		50	64	58	65	73	63	83	105	99	108	20	20	22	119	121	137	23	23	24
		60	63	64	80	62	58	61	97	103	120	21	20	21	115	130	124	24	23	23
		70	31	34	37	91	97	109	114	119	119	25	25	24	74	107	109	30	31	29
		80	23	40	36	61	62	80	65	77	85	25	25	25	26	105	112	30	30	30
		90	17	52	47	89	83	145	63	69	87	28	24	22	7	95	105	33	31	28
		100	21	22	31	52	89	119	73	99	102	28	23	19	44	99	108	35	27	23

0-20%	20-50%	50-70%	70-100%	100-120%	120-150%

Table S6. Enzyme inhibition of fractions obtained after further separation of the extracts VIII and IX. Results are expressed as a percentage value of enzyme inhibition compared to untreated control. Different colors highlight the differences in values (the color code is explained below the table).

Enzymes			Trypsin		Chymotrypsin		Thrombin	
Conc., ug/mL			45	4.5	45	4.5	45	4.5
Fractions	VIII	10	94	-	-	-	-	-
		20	73	-	99	-	95	-
		30	58	-	-	-	-	-
		40	8	21	54	-	15	80
		50	7	65	10	66	15	40
		60	7	61	9	26	11	90
		70	8	20	9	20	11	95
		80	9	17	11	69	41	90
		90	10	25	43	92	36	80
		100	13	87	34	88	67	-
	IX	10	68	-	-	-	-	-
		20	-	-	-	-	-	-
		30	8	96	95	-	88	-
		40	8	16	29	92	18	95
		50	7	54	10	72	10	21
		60	7	32	11	40	35	85
		70	7	50	10	41	15	87
		80	9	12	11	69	18	41
		90	9	39	34	90	88	-
		100	16	95	50	95	-	-

High enzymatic inhibition			Low enzymatic inhibition	
0-20%	20-50%	50-70%	70-100%	100-120%

Table S7. T47D cancer cells viability of fractions obtained after further separation of the extracts IV, and fractions IV-50, IV-60, IV-70. Results are expressed as a range of percentage effect compared to untreated control. Different colors have been applied to highlight the differences in values (the color code is explained below the table).

Cancer cell line			T47D			
Conc., ug/ml			200	100	50	25
Fractions	IV	10	84	87	82	88
		20				
		30	88	97	93	104
		40	90	99	89	94
		50	54	69	70	87
		60	3	87	80	-
		70	36	57	53	47
		80	87	82	76	86
		90	79	79	80	89
		100	91	95	95	91
	IV-50	20	111	100	109	111
		40	112	101	109	108
		60	99	85	85	92
		90	102	90	96	102
		100	83	77	90	94
	IV-60	20	119	98	95	86
		40	88	85	90	97
		60	78	74	83	89
		90	79	71	75	81
		100	89	86	101	101
	IV-70	20	58	72	102	105
		40	108	104	94	103
		60	97	87	90	91
		90	22	81	88	91
		100	94	90	92	93

Low cell viability		High cell viability		
0-20%	20-50%	50-70%	70-100%	100-120%

Table S8. Presence and dominance of phytoplankton species in the samples (1-9) from the Curonian lagoon. Cyanobacteria dominance was expressed as a percentage of total cyanobacteria biomass. The dominance of other species of phytoplankton was expressed as a percentage of total phytoplankton biomass. The color code is explained below the table.

**Cyanophyceae**	1	2	3	4	5	6	7	8	9
Aphanocapsa sp.									
Aphanocapsa holsatica									
Aphanothece minutissima									
Aphanocapsa delicatissima									
Aphanocapsa incerta									
Aphanocapsa planctonica									
Aphanocapsa stagnalis									
Aphanothece sp.									
Anathece clatrata									
Anathece minutissima									
Anabaena sp.									
Anabaena planctonica									
Anabaena cylindrica									
Anabaena mendotea									
Anabaena spiroides									
Dolichospermum crassum									
Dolichospermum planctonicum									
Dolichospermum flosaquae									
Dolichospermum lemmermanii									
Aphanizomenon flosaquae									
Aphanizomenon gracile									
Cuspidothrix issatschenkoi									
Chroococcus dispersus									
Chroococcus turgidus									
Chroococcus minimus									
Coelomoron pussilum									
Limnococcus limneticus									
Cyanodictyon imperfectum									
Cyanodictyon planctonicum									
Chroococcus aphanocapsoides									
Coelosphaerium kuetzingianum									
Coelomoron pussilum									
Limnothrix redekei									
Merismopedia sp.									
Merismopedia warmingiana									
Merismopedia punctata									
Merismopedia tenuissima									
Microcystis sp.									
Microcystis flos-aquae									
Microcystis aeruginosa									

**Cyanophyceae**	1	2	3	4	5	6	7	8	9
Microcystis viridis									
Microcystis wesenbergii									
Planktothrix agardhii									
Pseudanabaena sp.									
Pseudanabaena limnetica									
Pseudanabaena mucicola									
Planktolyngbya sp.									
Planktolyngbya limnetica									
Radiocystis geminata									
Romeria elegans									
Woronichinia compacta									
Woronichinia naegeliana									

**Bacillariophyceae**	1	2	3	4	5	6	7	8	9
Melosira sp.									
Achnanthidium minutissimum									
Aulacoseira islandica ssp. helvetica									
Aulacoseira islandica ssp. islandica									
Aulacoseira granulata v. granulata									
Actinocyclus normanii									
Asterionella formosa									
Surirella biseriata									
Synedra acus v. angustissima									
Cyclotella sp.									
Cyclotella glomerata									
Cymatopleura solea									
Cymbella sp.									
Diatoma tenuis									
Fragilaria capucina v. capucina									
Fragilaria crotonensis									
Gyrosigma attenuatum									
Navicula capitata									
Navicula cryptocephala									
Navicula sp.									
Nitzschia recta									
Nitzschia acicularis v. acicularis									
Skeletonema subsalsum									
Staurosira construens v. construens									
Staurosira construens v. binodis									

**Bacillariophyta**	1	2	3	4	5	6	7	8	9
Staurosira construens v. venter									
Stephanodiscus hantzschii									
Stephanodiscus rotula									
Surirella biseriata									
Surirella robusta									
Surirella elegans									
Synedra berolinensis									
Synedra acus v. angustissima									
Synedra acus v. acus									
Synedra ulna v. ulna									

**Chlorophyta**	1	2	3	4	5	6	7	8	9
Ankistrodesmus fusiformis									
Closterium aciculare									
Closterium acutum									
Closterium acutum v. variabile									
Closterium acerosum									
Closterium gracile									
Closterium pronum									
Closterium strigosum									
Coelastrum astroideum									
Coelastrum reticulatum									
Coelastrum sphaericum									
Coelastrum microporum									
Crucigenia tetrapedia									
Crucigeniella rectangularis									
Chlorococcales sp.									
Chlamydomonas sp.									
Cosmarium sp.									
Desmodesmus abundans									
Desmodesmus armatus									
Desmodesmus brasiliensis									
Desmodesmus communis									
Desmodesmus maximus									
Desmodesmus opoliensis									
Desmodesmus subspicatus									
Dictyosphaerium ehrenbergianum									
Dictyosphaerium pulchellum									

**Chlorophyta**	1	2	3	4	5	6	7	8	9
Dictyosphaerium subsolitarium									
Ellakatothrix gelatinosa									
Elakatothrix genevensis									
Flagellates spp.									
Golenkinia radiata									
Kirchneriella contorta									
Korschikoviella limnetica									
Klebsormidium sp.									
Micractinium pusillum									
Monoraphidium contortum									
Monoraphidium griffithii									
Monoraphidium komarkovae									
Monoraphidium minutum									
Monactinus simplex									
Mougeotia sp.									
Oocystis sp.									
Oocystis borgei									
Oocystis parva									
Oocystis solitaria									
Oocystis rhomboidei									
Oocystis lacustris									
Oocystis marssonii									
Pandorina morum									
Pediastrum boryanum v. boryanum									
Pediastrum duplex v. duplex									
Pediastrum simplex									
Pediastrum kawraiskyi									
Planctonema lauterbornii									
Scenedesmus arcuatus									
Scenedesmus ellipticus									
Sphaerocystis schroeteri									
Schroederia setigera									
Staurastrum sp.									
Staurastrum planctonicum									
Lagerheimia subsalsa									
Lagerheimia ciliate									
Tetrachlorella alternans									
Tetrastrum staurogeniaforme									
Tetrastrum komarekii									

**Chlorophyta**	1	2	3	4	5	6	7	8	9
Treubaria triappendiculata									
Treubaria setigera									
Spirogyra sp.									
**Cryptophyta**	1	2	3	4	5	6	7	8	9
Komma caudata									
Rhodomonas lacustris									
Cryptomonas curvata									
Cryptomonas marssonii									
Cryptomonas ovata									
Rhodomonas lacustris									
Cryptomonadales									
**Dinophyta**	1	2	3	4	5	6	7	8	9
Perdidiniopsis polonica									
Ceratium hirundinella									
Gymnodinium sp.									
Gymnodinium simplex									
Gymnodiniales sp.									
Peridiniopsis polonica									
Peridiniopsis sp.									
Peridiniales sp.									
**Euglenophyta**	1	2	3	4	5	6	7	8	9
Trachelomonas sp.									
Trachelomonas volvocina									
Euglena viridis									
**Chrysophyta**	1	2	3	4	5	6	7	8	9
Mallomonas sp.									
Mallomonas acaroides									
**Haptophyta**	1	2	3	4	5	6	7	8	9
Chrysochromulina spp.									

**0%**	**0,001-5%**	**5-10%**	**10-20%**	**20-50%**	**50-100%**

Table S9. Microcystins (MCs) and other oligopeptides detected in the most active fractions obtained from the phytoplankton samples collected in the Curonian Lagoon (the numbers in parentheses are given to distinguish compounds with the same molar mass but different amino acid sequences).

Assays	Active fractions		Detected cyanometabolites and *m/z* (amino acids sequences are listed in Table S2)		Potentially toxic dominant cyanobacteria species (% from the total cyanobacteria biomass)
Antibacterial, Cytotoxicity	**IV**	**60**	**MCs:** MC-RR; MC-LR; Dha^{7}MC-RR<br>**CPs:** CP1023; CP989; CP986 (3); CP978 (2); CP970; CP951; CP965; CP916; CP902; CP886 (2)<br>**Micropeptin B**<br>**AERs:** AER604 (K139)<br>**APs:** AP F; AP848; AP B; Hph^{4}AP F<br>**MGs:** MG753; MG755; MG726 (FR5); MG713; MG642	**MC: 3**<br>**CP: 10**<br>**Micropeptin B**<br>**AER: 1**<br>**AP: 4**<br>**MG: 5**	*W. compacta* (33.10%), *Aph. flosaquae* (19.06%), *M. wesenbergii* (6.88%), *D. lemmermannii* (6.77%), *D. crassum* (2.88%), *A. cylindrica* (2.65%), *M. viridis* (2.62%)
		**70**	**MCs:** MC-LF; MC-RR; Asp^{3}Dha^{7}MC-Rhty<br>**CPs:** CP1037; CP1055; CP1014; CP1006 (1); CP993; CP989; CP986 (2); CP978 (1); CP972; CP962; CP960; CP916; CP902<br>**AER:** AER636; AER604 (K139)<br>**MGs:** MG928; MG783; KR767; MG753; MG755; MG748; MG743; MG741; MG728 (FR1); MG727 (FR3); MG713; MG678	**MC: 3**<br>**CP: 13**<br>**AER: 2**<br>**MG: 12**	
		**80**	**CPs:** CP1006 (2)	**CP: 1**	
		**90**	**Unidentified**	**-**	
		**100**	**CPs:** CP986 (2)	**CP: 1**	
Cytotoxicity	**IV-70**	**90**	**MCs:** MC-RR<br>**CPs:** CP1048; CP1015; CP1014; CP1006 (1); CP1006 (2); CP992; CP986 (2); CP978 (1); CP972; CP962; CP960; CP916; CP886 (1)<br>**Micropeptin B**<br>**AERs:** AER636; AER526; AER604 (K139)<br>**MGs:** MG928; MG783; MG769; MG768 (KR767); MG753; MG743; MG741; MG729 (FR1); MG727 (FR3); MG726 (FR5); MG713; MG678; MG650	**MC: 1**<br>**CP: 13**<br>**Micropeptin B**<br>**AER: 3**<br>**MG: 13**	
Antibacterial	**V**	**70**	**MCs:** MC-RR; Dha^{7}MC-RR<br>**CPs:** CP1045; CP1023; CP1015; CP1012; CP1006 (1); CP993; CP989; CP962; CP946; CP916; CP902<br>**Micropeptin B**<br>**AERs:** AER604 (K139)<br>**APs:** AP848; Hph^{4}AP F<br>**MGs:** MG769; MG768 (KR767); MG753; MG755; MG741; MG727 (FR3); MG726 (FR5); MG642	**MC: 2**<br>**CP: 11**<br>**Micropeptin B**<br>**AER: 1**<br>**AP: 2**<br>**MG: 8**	*W. compacta* (32.31%), *M. wesenbergii* (10.40%), *M. viridis* (7.82%), *M. flosaquae* (7.01%), *Aph. flosaquae*, (6.30%), *M. aeruginosa* (4.32%)
		**80**	**MCs:** MC-RR<br>**CPs:** CP1012; CP1006 (1); CP1006 (2); CP992; CP986 (2); CP978 (1); CP962; CP960<br>**Micropeptin B**	**MC: 1**<br>**CP: 8**<br>**Micropeptin B**	
		**90**	**CPs:** CP1014; CP986 (2); CP972<br>**Micropeptin A**	**CP: 3**<br>**Micropeptin A**	
		**100**	**Unidentified**	**-**	
Enzyme inhibition	**VIII**	**40**	**MCs:** MC-LF<br>**CPs:** CP912; CP895; CP887<br>**AERs:** AER652; AER640; AER617; AER604 (K139)<br>**APs:** AP G; AP A	**MC: 1**<br>**CP: 3**<br>**AER: 4**<br>**AP: 2**	*W. compacta* (34.79%), *Aph. flosaquae* (10.75%), *M. viridis* (6.78%), *C. issatschenkoi* (6.59%), *M. wesenbergii* (4.84%), *M.s flosaquae* (3.96%), *D. crassum* (3.50%), *P. agardhii* (2.99%), *P. limnetica* (2.52%), *Aph. gracile* (2.00%)
		**50**	**MCs:** SerMC-HtyR; MC-WR; MC1049; MC-YR; MC-FR; MC-LF; MC-LR<br>**CPs:** CP986 (3); CP970; CP936; CP912; CP871<br>**AERs:** AER638; AER636; AER617; AER588; AER567<br>**APs:** AP885; AP884; AP871; OscY; AP A; AP B; AP D	**MC: 7**<br>**CP: 5**<br>**AER: 5**<br>**AP: 7**	
		**60**	**MCs:** MC1068; MC-RR; MC-LY; MC-HilR; MC-LR; Asp^{3}MC-LY; Dha^{7}MC-LR; Dha^{7}MC-RR.<br>**CPs:** CP1011; CP995; CP986 (3); CP984; CP978 (1); CP978 (2); CP970; CP962; CP951; CP946; CP939; CP936; CP930; CP916; CP902; CP895; CP887; CP886 (2)<br>**AERs:** AER638; AER617; AER604 (K139); AER588	**MC: 8**<br>**CP: 18**<br>**AER: 4**<br>**AP: 7**<br>**MG: 2**	

			**APs:** AP G; AP871; OscY; AP F; AP848; AP841; AP B<br>**MGs:** MG727 (FR3); MG713		
		70	**MCs:** MC-LW; MC-RR; MC-LR; Dha[7]MC-RR<br>**CPs:** CP1045; CP1012; CP995; CP986 (3); CP984; CP978 (2); CP951; CP936; CP916; CP902; CP886 (2)<br>**AERs:** AER638; AER636; AER618; AER617; AER604 (K139); AER588; AER567; AER552<br>**APs:** AP F; AP848<br>**MGs:** MG744; MG726 (FR5); MG713	**MC: 4**<br>**CP: 11**<br>**AER: 8**<br>**AP: 2**<br>**MG: 3**	
		80	**MCs:** MC1068; MC-HphR; MC-LW; MC-LF; MC-RR; MC-HilR; MC-LR; Dha[7]MC-LR; Dha[7]MC-RR<br>**CPs:** CP1037; CP1029; CP1055; CP1012; CP1006 (1); CP992; CP989; CP978 (2); CP960; CP944; CP916; CP902; CP886 (1)<br>**Micropeptin B**<br>**AERs:** AER638; AER636; AER618; AER617; AER588; AER567; AER552<br>**APs:** -<br>**MGs:** MG769; MG768 (KR767); MG753; MG755; MG748; MG744; MG743; MG741; MG729 (FR1); MG727 (FR3); MG713; MG650+H_2O	**MC: 9**<br>**CP: 13**<br>**Micropeptin B**<br>**AER: 7**<br>**AP: 0**<br>**MG: 12**	
		90	**MCs:** MC-RR; Asp[3]Dha[7]MC-Rhty; Asp[3]Dha[7]MC-YR; Dha[7]MC-RR<br>**CPs:** CP1020; CP1012; CP1006 (1); CP1006 (2); CP992; CP986 (2); CP978; CP962 (1); CP960<br>**Micropeptin B**<br>**AERs:** AER618; AER617; AER588; AER567<br>**MGs:** MG769; MG768 (KR767); MG753; MG755; MG748; MG743; MG741; MG729 (FR1); MG727 (FR3); MG726 (FR5); MG713	**MC: 4**<br>**CP: 9**<br>**Micropeptin B**<br>**AER: 4**<br>**MG: 11**	
		100	**MCs:** MC-RR; Asp[3]Dha[7]MC-Rhty<br>**CPs:** CP972<br>**AERs:** AER618; AER617; AER567	**MC: 2**<br>**CP: 1**<br>**AER: 3**	
**Enzyme inhibition**	**IX**	30	**Unidentified**	**-**	*Aph. flosaquae* (38.59%), *D. crassum* (11.24%), *M. wesenbergii* (10.87%), *M. viridis* (7.44%), *M. flosaquae* (7.01%), *A. spiroides* (5.76%), *W. compacta* (3.85%), *D. flosaquae* (3.66%), *C. issatschenkoi* (2.96%), *Aph. gracile* (2.69%), *P. agardhii* (1.74%).
		40	**CPs:** CP912; CP871<br>**AERs:** AER617<br>**APs:** AP A	**CP: 2**<br>**AER: 1**<br>**AP: 1**	
		50	**MCs:** MC-YR; Asp[3]Dha[7]MC-Rhty; MC-LR<br>**AERs:** AER617; AER567<br>**APs:** OscY; AP A	**MC: 3**<br>**AER: 2**<br>**AP: 2**	
		60	**MCs:** MC1068; MC-RR; MC-LR; Dha[7]MC-LR<br>**CPs:** CP995; CP978 (2); CP965; CP902; CP895; CP886 (1); CP886 (2)<br>**AERs:** AER617; AER588; AER567<br>**APs:** AP F; AP B<br>**MGs:** MG727 (FR3)	**MC: 4**<br>**CP: 7**<br>**AER: 3**<br>**AP: 2**<br>**MG: 1**	
		70	**MCs:** MC-RR; Dha[7]MC-RR<br>**CPs:** CP1015; CP1012; CP1009; CP993; CP965; CP916; CP902<br>**AERs:** AER638; AER604 (K139); AER588; AER567<br>**APs:** AP848; Hph[4]AP F<br>**MGs:** MG753; MG741; MG727 (FR3); MG726 (FR5); MG713	**MC: 2**<br>**CP: 7**<br>**AER: 4**<br>**AP: 2**<br>**MG: 5**	
		80	**MCs:** MC-HphR; MC-RR<br>**CPs:** CP1029; CP1023; CP1015; CP1006 (1); CP992; CP989; CP978 (1); CP962<br>**Micropeptin B**<br>**AERs:** AER617; AER604 (K139); AER567<br>**MGs:** MG769; MG768 (KR767); MG753; MG748; MG741; MG727 (FR3)	**MC: 2**<br>**CP: 8**<br>**Micropeptin B**<br>**AER: 3**<br>**MG: 6**	
		90	**MCs:** Asp[3]Dha[7]MC-Rhty<br>**CPs:** CP1020; CP1006 (2); CP978 (1); CP972<br>**AERs:** AER567	**MC: 1**<br>**CP: 4**<br>**AER: 1**	
		100	**MCs:** Asp[3]Dha[7]MC-Rhty<br>**CPs:** CP986 (2)<br>**AERs:** AER617; AER567	**MC: 1**<br>**CP: 1**<br>**AER: 2**	

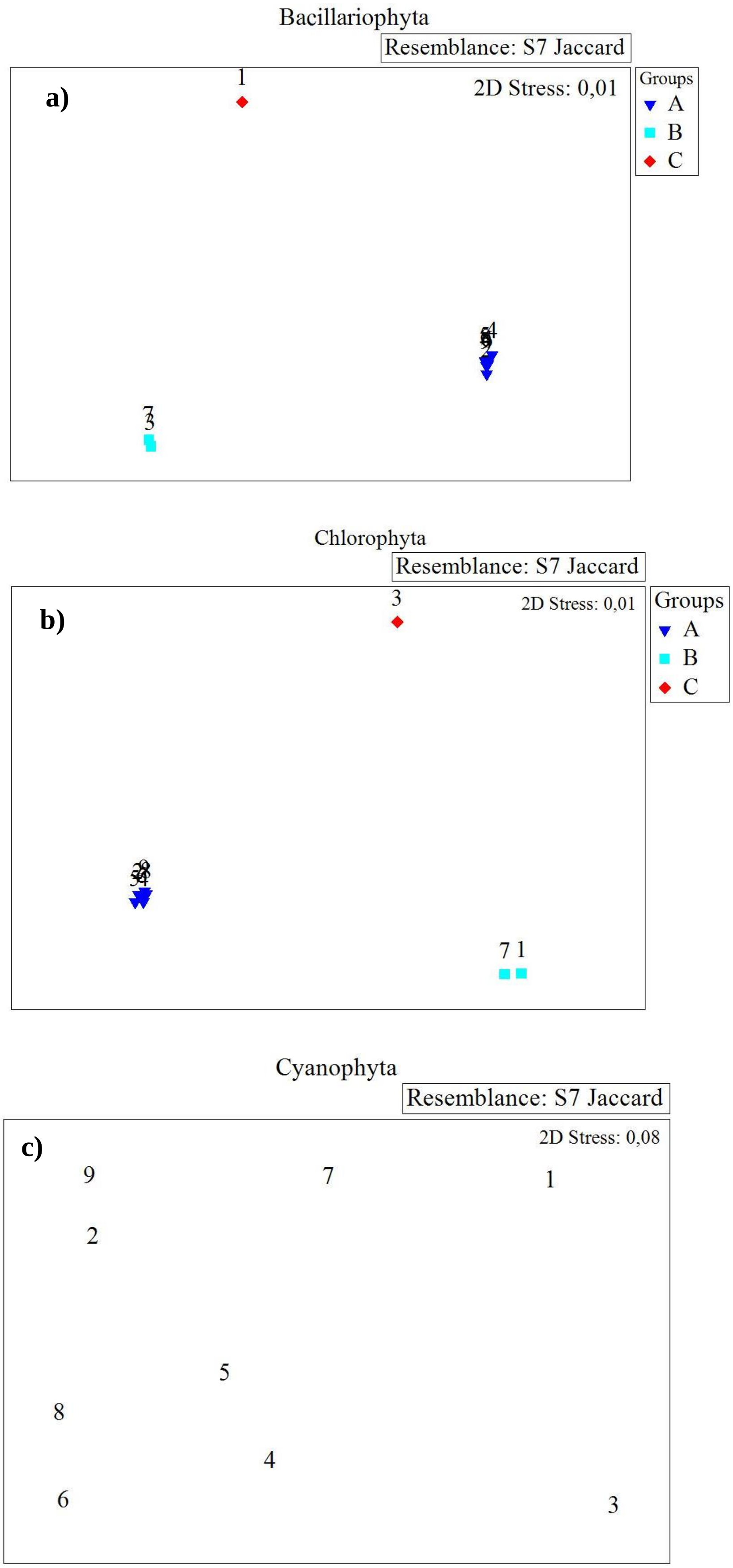

Figure S1. nMDS plot based on presence/absence Jaccard similarity matrix of Bacillariophyta (a), Chlorophyta (b), and Cyanophyta (c) communities.

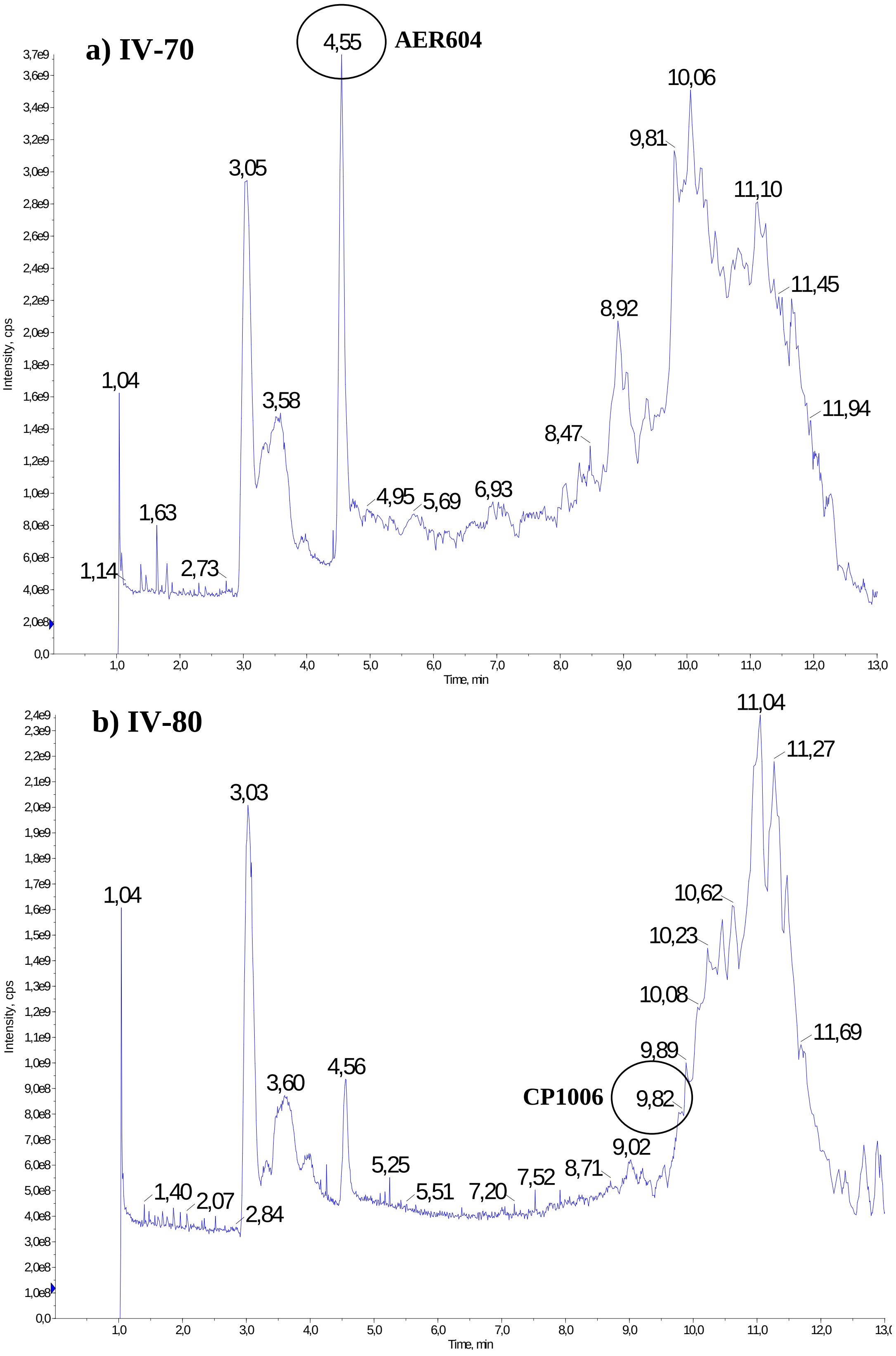
a) IV-70
4,55
AER604
Intensity, cps
Time, min
1,04
1,14
1,63
2,73
3,05
3,58
4,95
5,69
6,93
8,47
8,92
9,81
10,06
11,10
11,45
11,94
b) IV-80
1,04
1,40
2,07
2,84
3,03
3,60
4,56
5,25
5,51
7,20
7,52
8,71
9,02
CP1006
9,82
9,89
10,08
10,23
10,62
11,04
11,27
11,69
Intensity, cps
Time, min

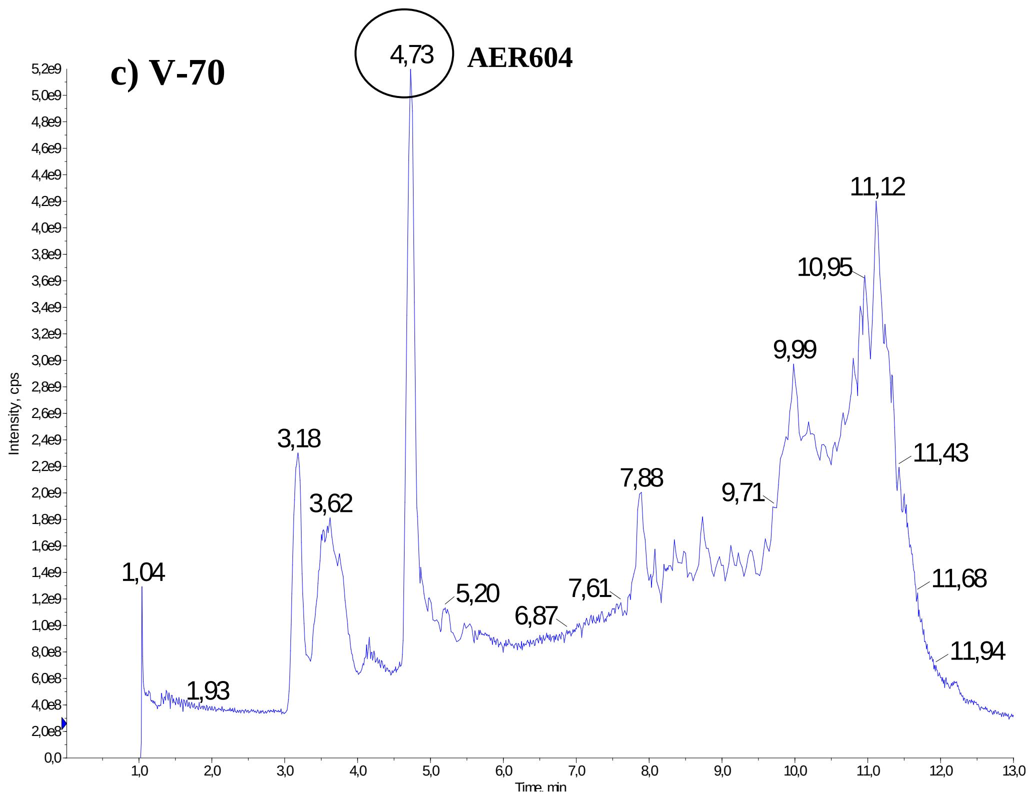
c) V-70
4,73
AER604
Intensity, cps
Time, min
1,04
1,93
3,18
3,62
5,20
6,87
7,61
7,88
9,71
9,99
10,95
11,12
11,43
11,68
11,94

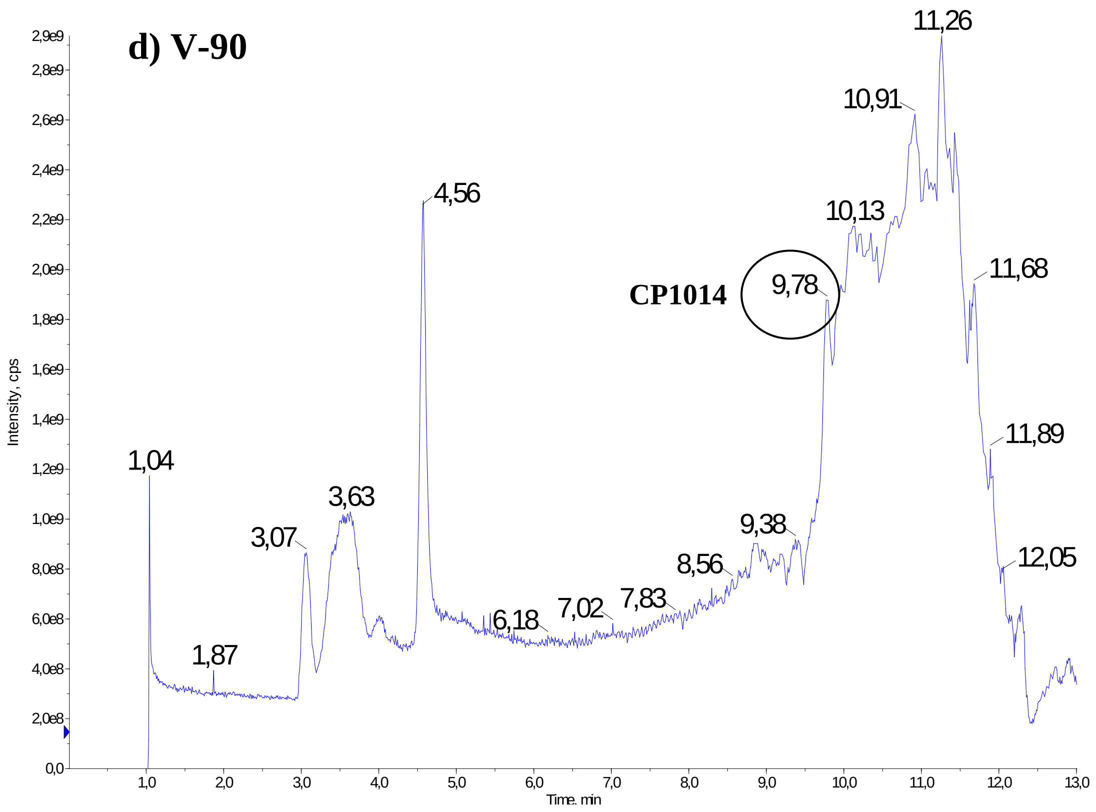
d) V-90
Intensity, cps
Time, min
1,04
1,87
3,07
3,63
4,56
6,18
7,02
7,83
8,56
9,38
CP1014
9,78
10,13
10,91
11,26
11,68
11,89
12,05

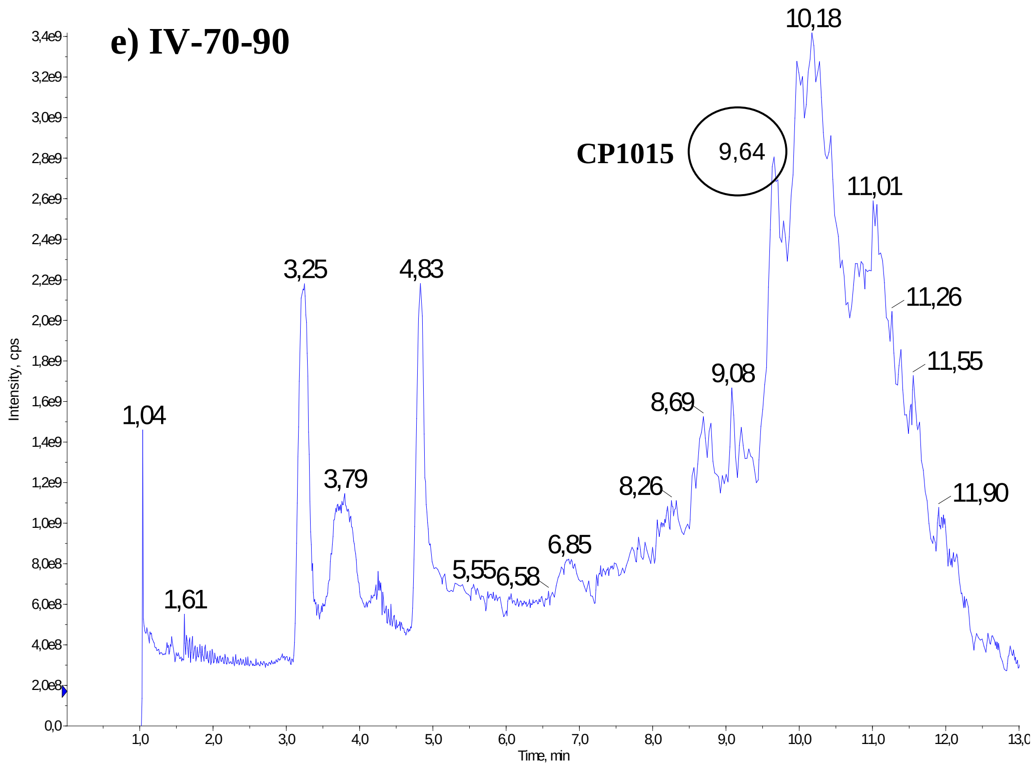
e) IV-70-90
CP1015
9,64
10,18
11,01
11,26
11,55
11,90
9,08
8,69
8,26
6,85
5,55
6,58
3,25
4,83
3,79
1,04
1,61
Intensity, cps
Time, min

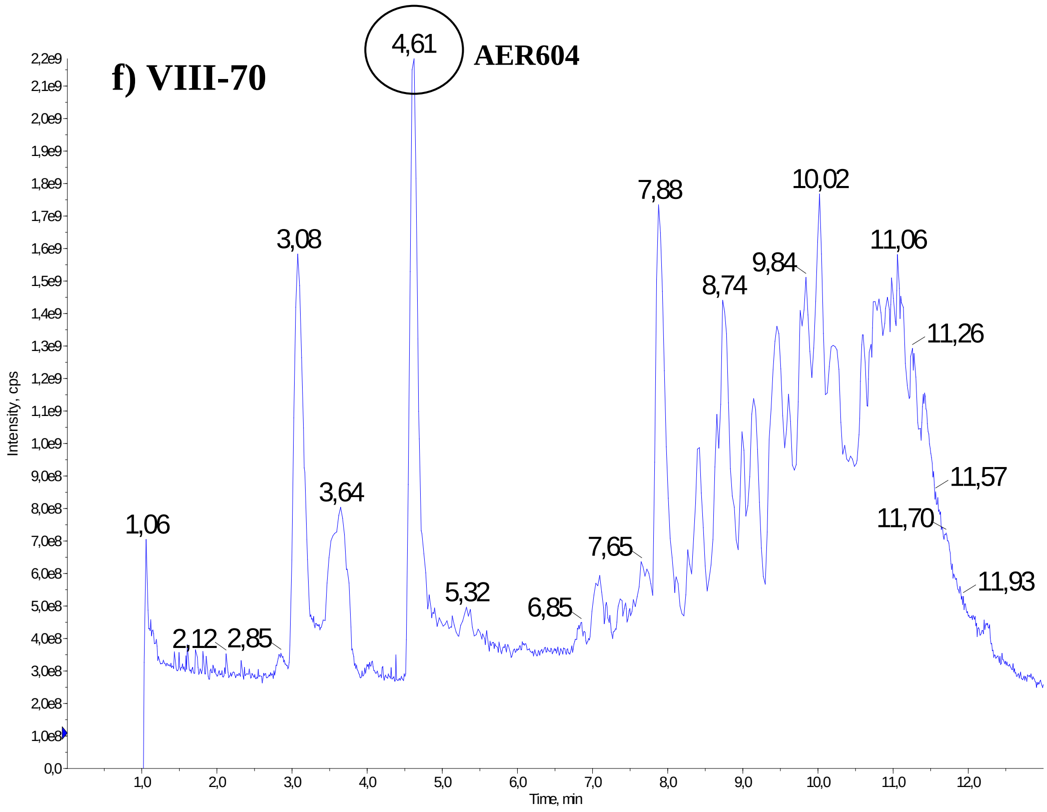
f) VIII-70
4,61
AER604
7,88
10,02
3,08
11,06
9,84
8,74
11,26
11,57
11,70
11,93
3,64
1,06
7,65
5,32
6,85
2,12
2,85
Intensity, cps
Time, min

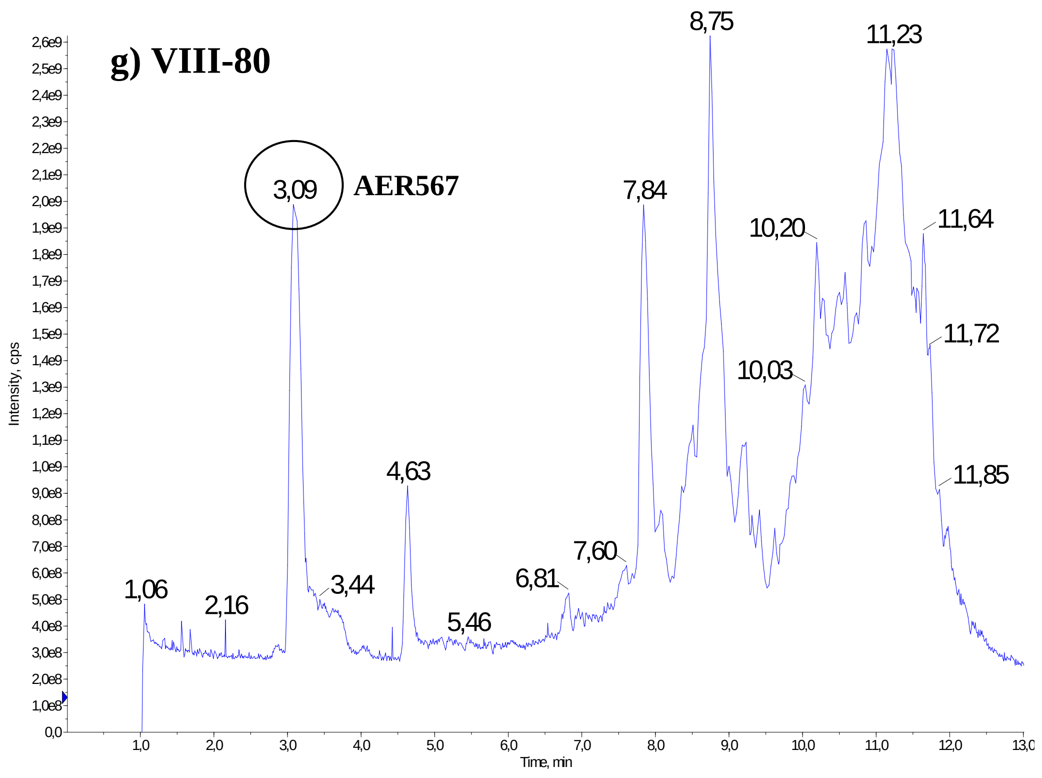
g) VIII-80
AER567
3,09
8,75
11,23
7,84
10,20
11,64
11,72
10,03
11,85
4,63
7,60
6,81
1,06
2,16
3,44
5,46
Intensity, cps
Time, min

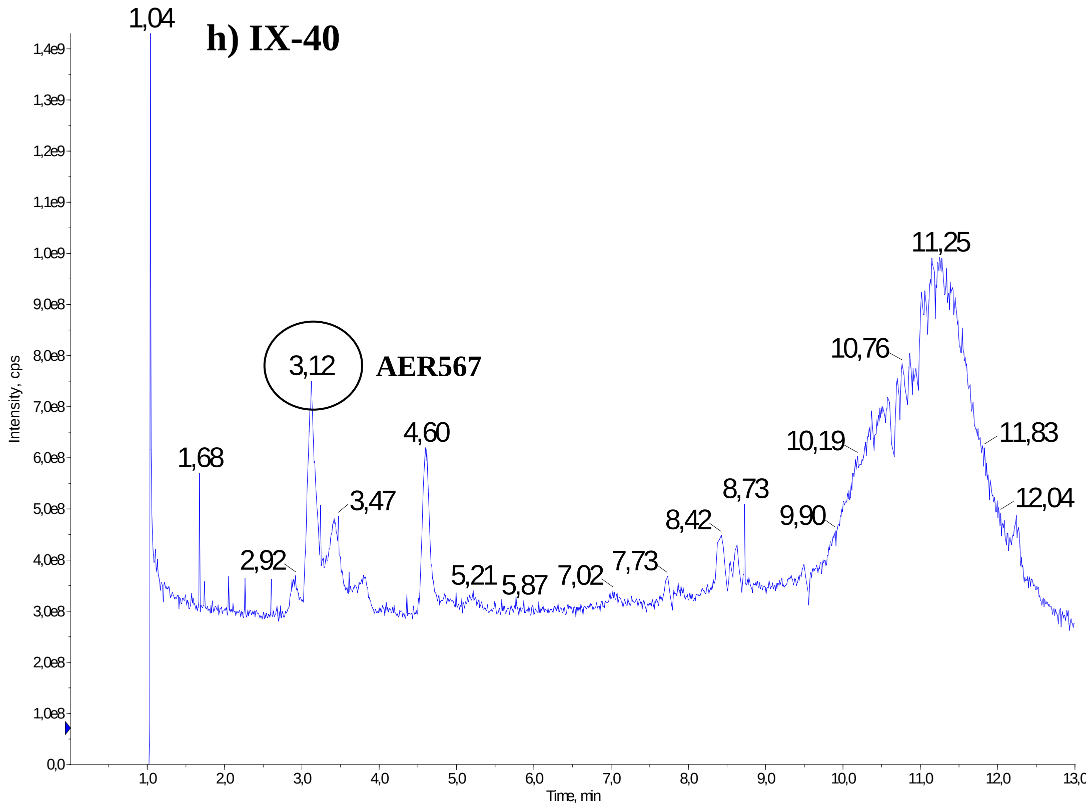
h) IX-40
1,04
AER567
3,12
11,25
10,76
10,19
11,83
4,60
1,68
3,47
8,73
12,04
8,42
9,90
2,92
7,73
5,21
5,87
7,02
Intensity, cps
Time, min

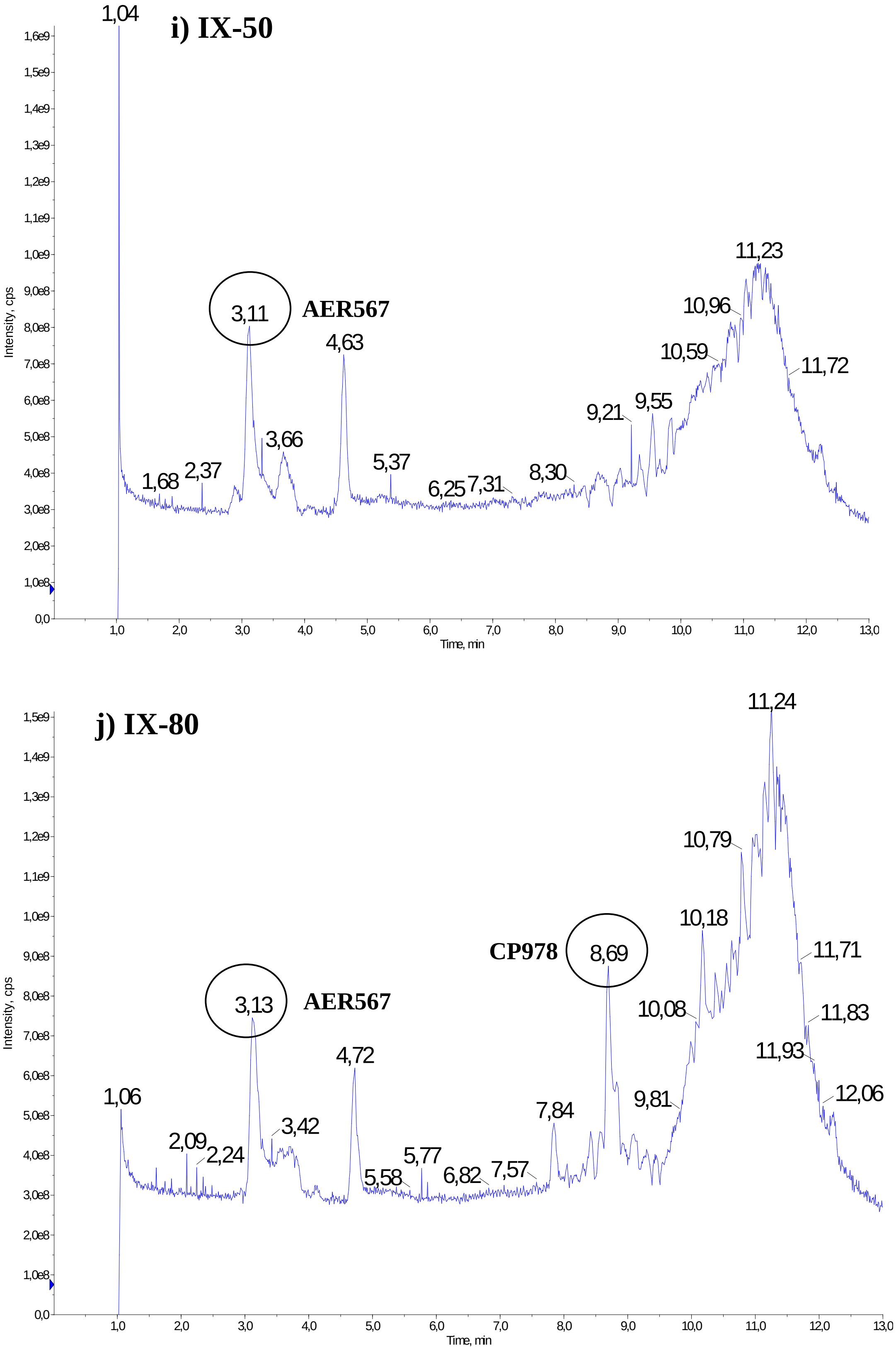

Figure S2. The total ion current (TIC) spectra of fractions from phytoplankton samples: IV-70 (a), IV-80 (b), V-70 (c), V-90 (d), IV-70-90 (e), VIII-70 (f), VIII-80 (g), IX-40 (h), IX-50 (i), IX-80 (j)

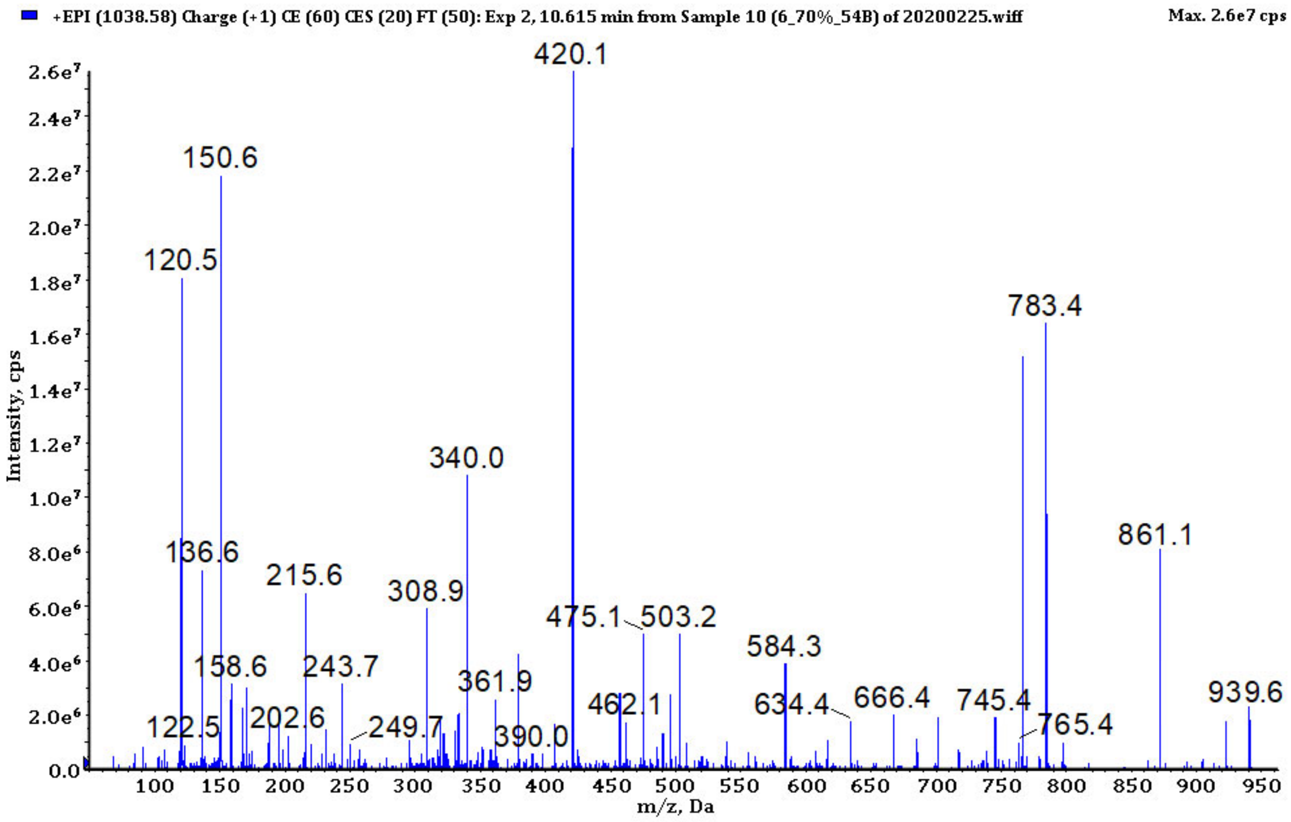

Figure S3. The enhanced product ion mass spectrum of cyanopeptoline CP1055 with the suggested structure OA + Gln – [Thr[1] + Tyr[2] + Ahp[3] + Phe[4] + MeTyr[5] + Val[6]] and the following fragment ions: *m/z* 939 [M + H – H_2O – Val]$^+$, 861 [M + H – H_2O – MeTyr]$^+$, 783 [M + H – H_2O – (OA + Gln)]$^+$, 765 [M + H – $2H_2O$ – (OA + Gln)]$^+$, 666 [M + H – $2H_2O$ – (OA + Gln) – Val]$^+$, 420 [Ahp + Phe + MeTyr + H – H_2O]$^+$, 150 MeTyr immonium; 136 Tyr immonium; 120 Phe immonium (OA – octanoic acid).

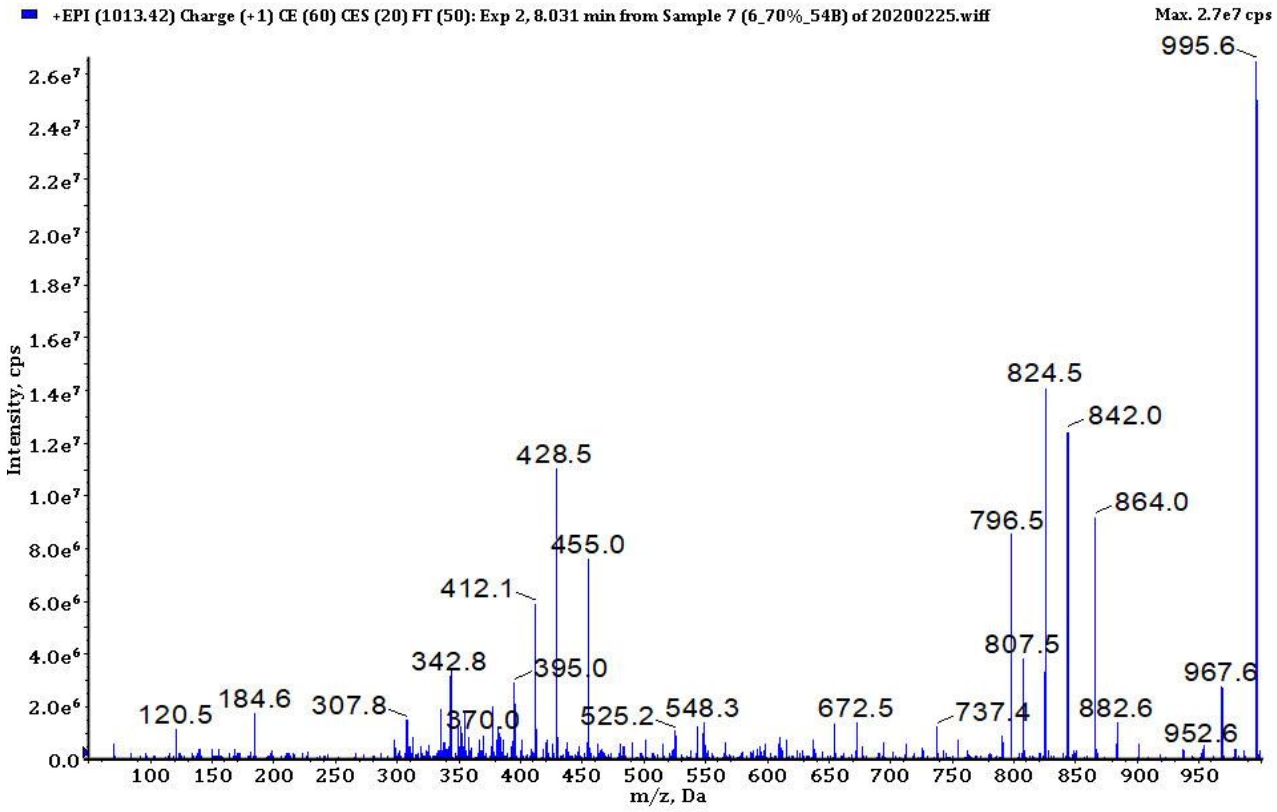

Figure S4. The enhanced product ion mass spectrum of cyanopeptoline CP1012 with the suggested structure Ac + Gln – [Thr[1] + Arg[2] + Ahp[3] + Phe[4] + ClMeTyr[5] + Ile[6]] and the following fragment ions: *m/z* 1013 [M + H]$^+$, 995 [M + H – H_2O]$^+$, 967 [M + H – H_2O – CO]$^+$, 882 [M + H – H_2O – Ile]$^+$, 864 [M + H – $2H_2O$ – Ile]$^+$, 842 [M + H – H_2O – (Ac + Gln)]$^+$, 824 [M + H – $2H_2O$ – (Ac + Gln)]$^+$, 796 [M + H – $2H_2O$ – (Ac + Gln) – CO]$^+$, 455 [Ahp + Phe + ClMeTyr + H – H_2O]$^+$, 428 [Ac + Gln + Thr + Arg + H]$^+$, 184 ClMeTyr immonium; 120 Phe immonium (Ac – acetyl group).

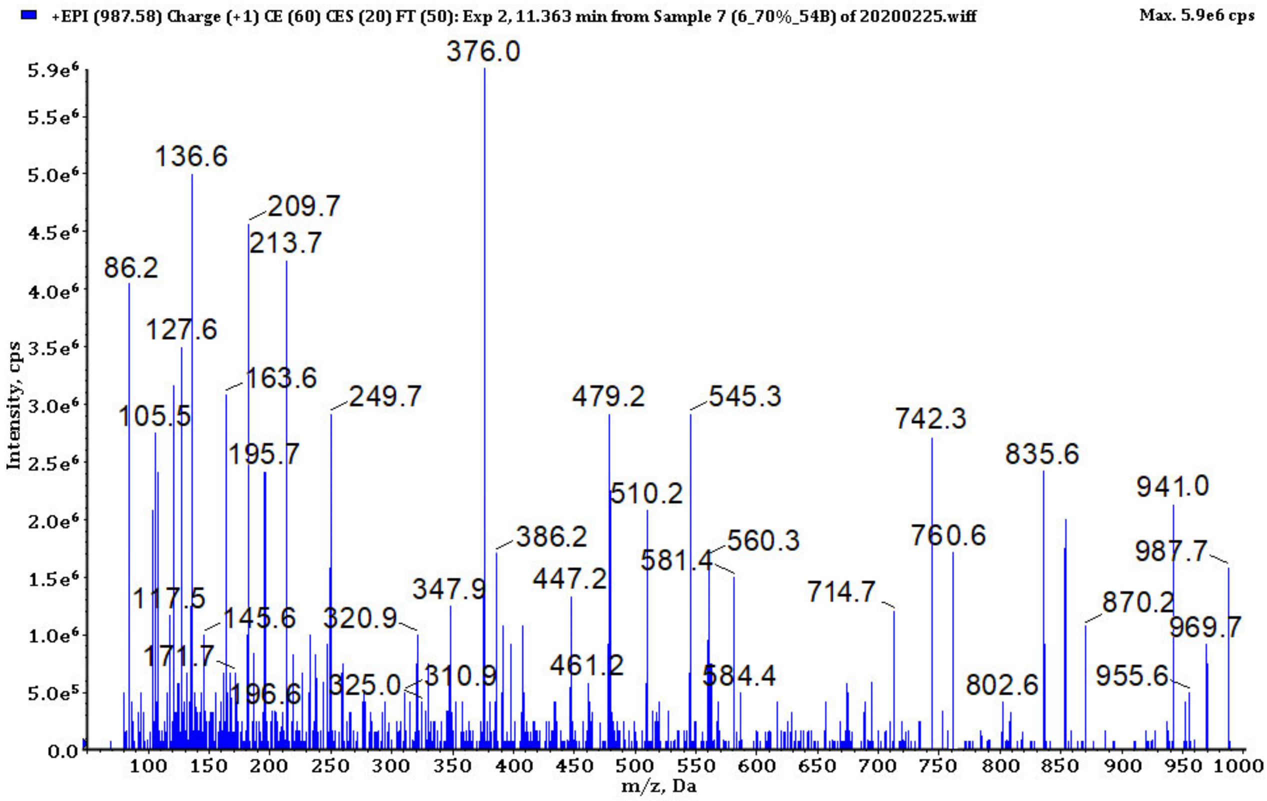

Figure S5. The enhanced product ion mass spectrum of cyanopeptoline CP986 with the suggested structure HA + Gln – [Thr^1 + Arg^2 + Ahp^3 + Leu^4 + MeTyr^5 + Val^6] and the following fragment ions: *m/z* 987 $[M + H]^+$, 969 [M + H – H_2O]+, 941 $[M + H – H_2O – CO]^+$, 870 $[M + H – HA – H_2O]^+$, 760 $[M + H – (HA + Gln)]^+$, 742 $[M + H – (HA + Gln) – H_2O]^+$, 714 $[M + H – (HA + Gln) – H_2O – CO]^+$, 584 $[M + H – (Ahp + Leu + MeTyr)]^+$, 484 $[HA + Gln + Thr + Arg + H]^+$, 386 $[Ahp + Ile + MeTyr + H – H_2O]^+$, 209 $[Ahp + Ile + H – H_2O]^+$, 181 $[Ahp + Ile + H – H_2O – CO]^+$, 150 MeTyr immonium, 136 Tyr immonium, 86 Ile immonium (HA – hexanoic acid).

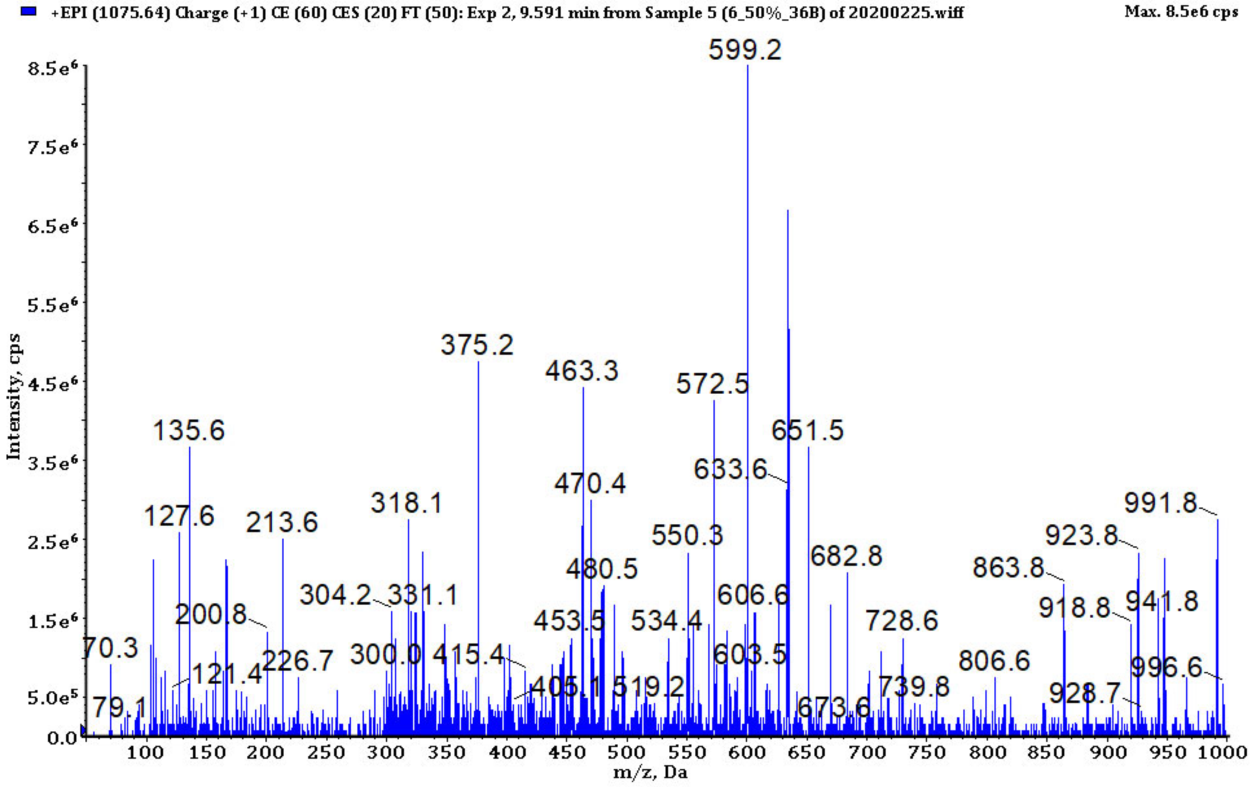

Figure S6. The enhanced product ion mass spectrum of microcystin [Ser^1]MC – HTyrR with the structure Adda – [Glu + Mdha + Ser + HTyr + MeAsp + Arg] and the following fragment ions: *m/z* 941 $[M + H – Adda]^+$, 923 $[C_{11}H_{14}O + Glu + Mdha + Ser + HTyr + MeAsp + Arg + H]^+$, 918 $[Adda + Glu + Mdha + Ser + HTyr + MeAsp + H]^+$, 863 $[Ser + HTyr + MeAsp + Arg + Adda + H]^+$, 728 $[MeAsp + Arg + Adda + Glu + H]^+$, 682 $[Arg + Adda + Glu + Mdha + H]^+$, 633 $[Mdha + Ser + HTyr + MeAsp + Arg + H]^+$, 599 $[Arg + Adda + Glu + H]^+$, 550 $[Ser + HTyr + MeAsp + Arg + H]^+$, 470 $[Arg + Adda + H]^+$, 375 $[C_{11}H_{14}O + Glu + Mdha + H]^+$, 213 $[Glu + Mdha + H]^+$, 135 Adda fragment.

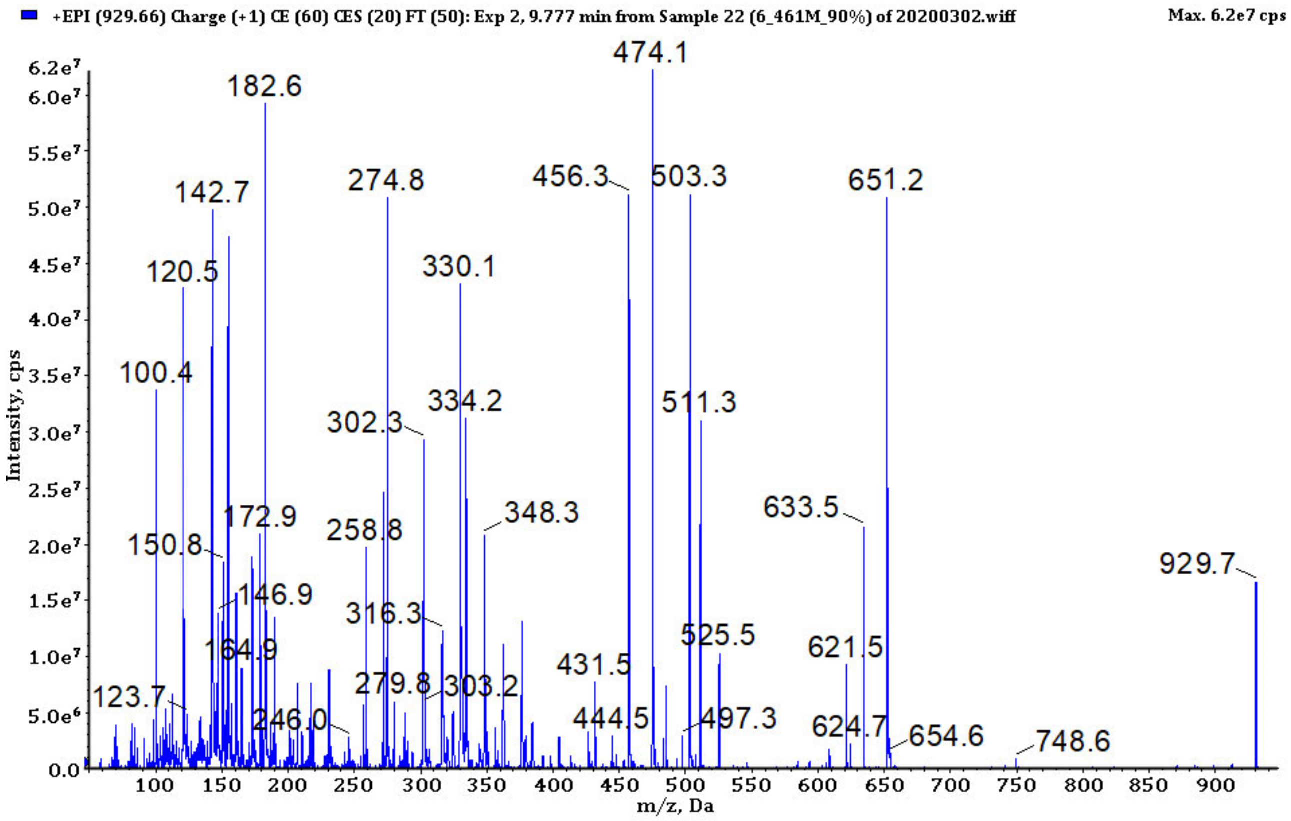

Figure S7. The enhanced product ion mass spectrum of microginin MG928 with the suggested structure MeAhda + Phe + MeLeu + HTyr + Pro + Tyr and the following fragment ions: *m/z* 929 $[M + H]^+$, 748 $[M + H – Tyr]^+$, 651 $[M + H – (Tyr + Pro)]^+$, 633 $[M + H – (Pro + Tyr) – H_2O]^+$, 474 $[M + H – (HTyr + Pro + Tyr)]^+$, 456 $[M + H – (HTyr + Pro + Tyr) – H_2O]^+$, 348 $[MeAhda + Phe + H]^+$, 330 $[MeAhda + Phe + H – H_2O]^+$, 182 $[MeAhda – H_2O]^+$, 172 $[MeAhda – CO]^+$, 150 Hty immonium ion, 142 MeAhda fragment, 120 Phe immonium, 100 MeLeu immonium.

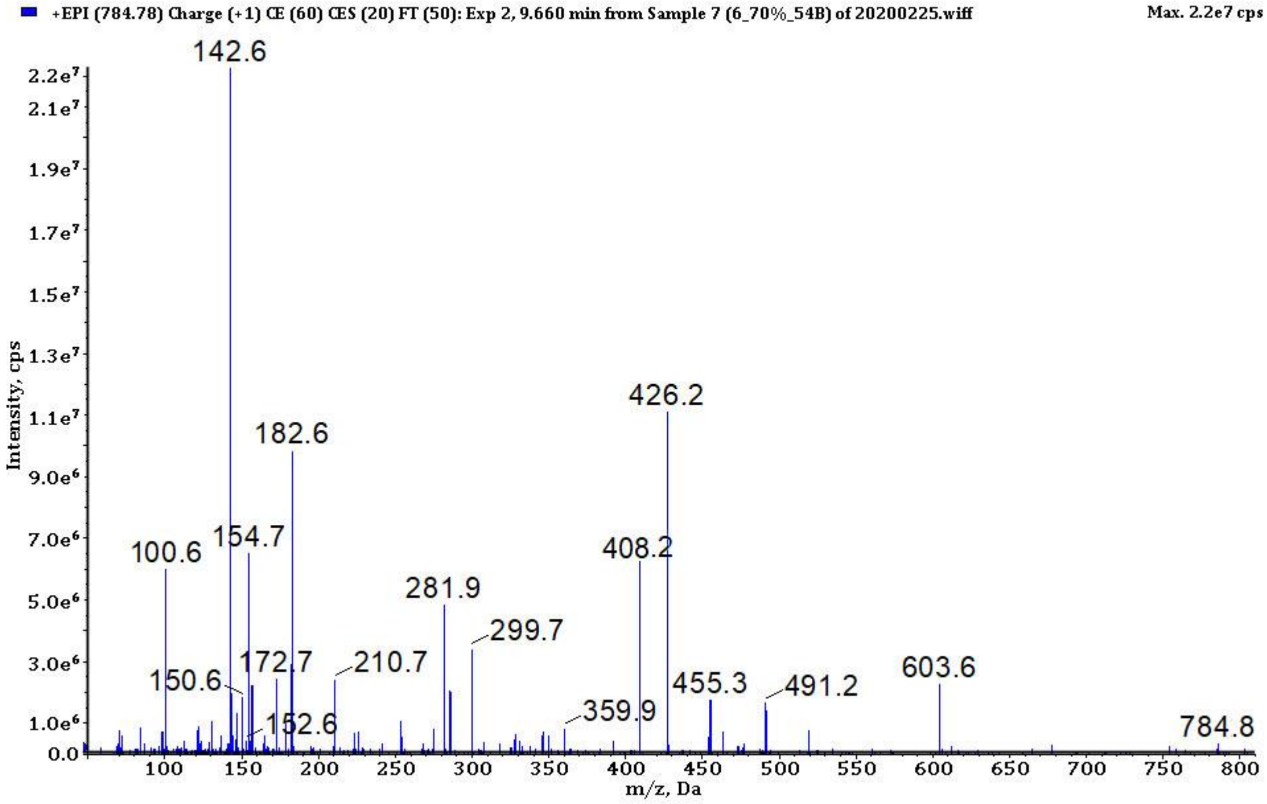

Figure S8. The enhanced product ion mass spectrum of microginin MG783 with the suggested structure MeAhda + Val + MeLeu + HTyr + Tyr and the following fragment ions: *m/z* 784 $[M + H]^+$, 603 $[M + H – Tyr]^+$, 426 $[M + H – (HTyr + Tyr)]^+$, 408 $[M + H – (HTyr + Tyr) – H_2O]^+$, 299 $[M + H – (MeLeu + HTyr + Tyr)]^+$, 281 $[M + H – (MeLeu + HTyr + Tyr) – H_2O]^+$, 182 $[MeAhda – H_2O]^+$, 172 $[MeAhda – CO]^+$, 142 MeAhda fragment, 100 MeLeu.

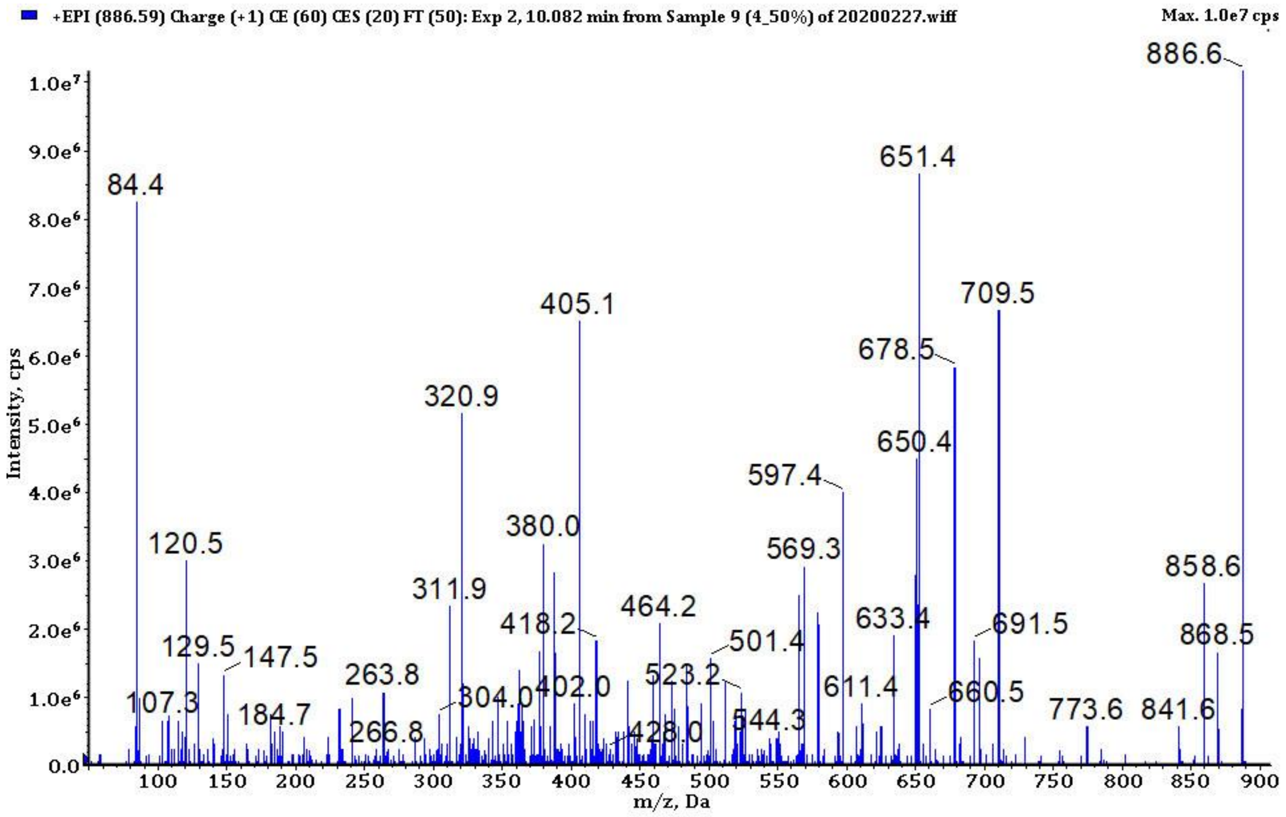

Figure S9. The enhanced product ion mass spectrum of anabaenopeptin AP885CL with the suggested structure MeHTyr + CO – [Lys + Ile + Hty + MeAla + Phe] and the following fragment ions: *m*/*z* 886 [M + H]$^+$, 868 [M + H – H_2O]$^+$, 858 [M + H – CO]$^+$, 773 [M + H – Ile]$^+$, 709 [M + H – HTyr]$^+$, 691 [M + H – HTyr – H_2O]$^+$, 651 [M + H – (CO + MeHTyr)]$^+$, 633 [M + H – (CO + MeHTyr) – H_2O]$^+$, 405 [M + H – MeHTyr – (Hty + Ile)]$^+$, 320 [M + H – MeHTyr – (MeAla + Hty + Ile)]$^+$, 263 [MeAla + Hty + H]$^+$, 120 Phe immonium, 107 Tyr/HTyr, 84 Lys.

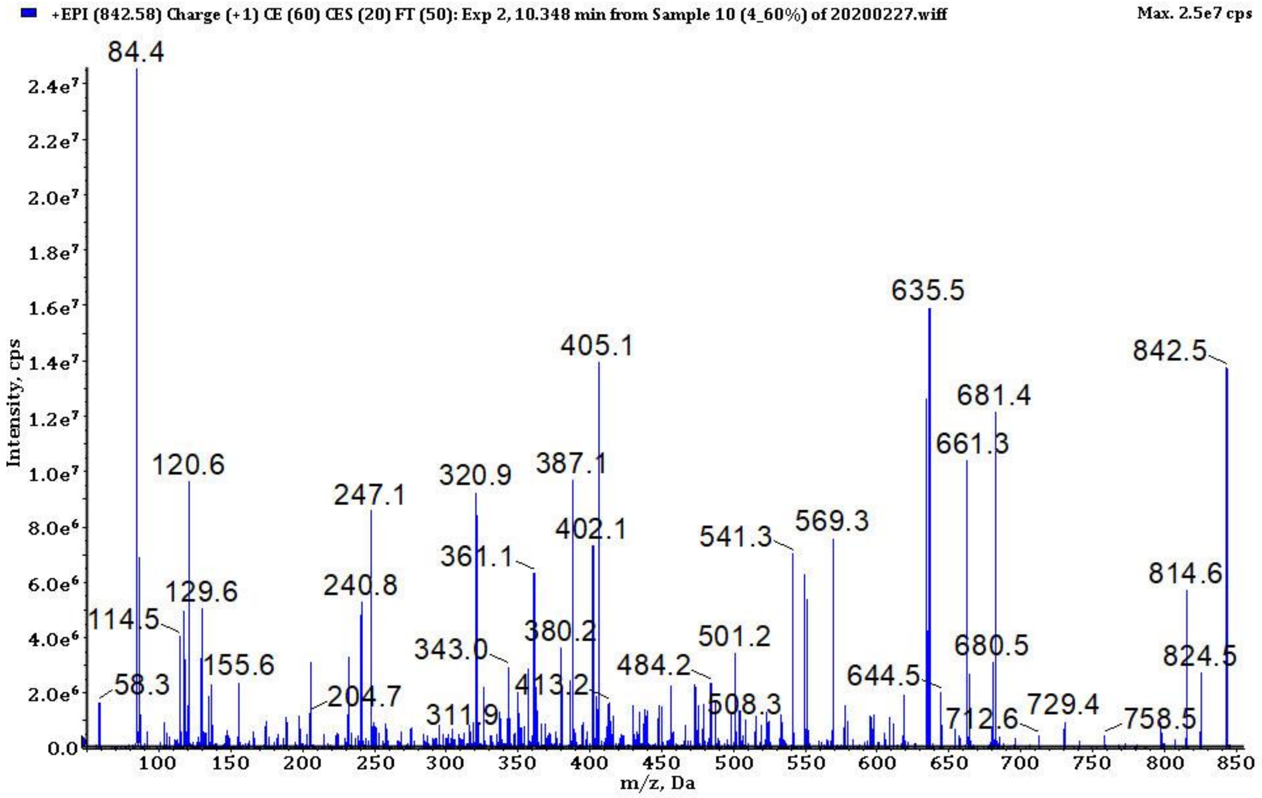

Figure S10. The enhanced product ion mass spectrum of anabaenopeptin AP841CL with the suggested structure Tyr + CO – [Lys + Ile + Hph + MeAla + Phe] and the following fragment ions: *m*/*z* 842 [M + H]$^+$, 824 [M + H – H_2O]$^+$, 814 [M + H – CO]$^+$, 729 [M + H – Ile]$^+$, 681 [M + H – Hph]$^+$, 661 [M + H – Tyr – H_2O]$^+$, 635 [M + H – (CO + Tyr)]$^+$, 405 [M + H –Tyr – (Hph + Ile)]$^+$, 387 [M + H – Tyr – (Hph + Ile) – H_2O]$^+$, 320 [M + H – Tyr – (MeAla + Hph + Ile)]$^+$, 247 [MeAla + Hph + H]$^+$, 120 Phe immonium, 84 Lys.

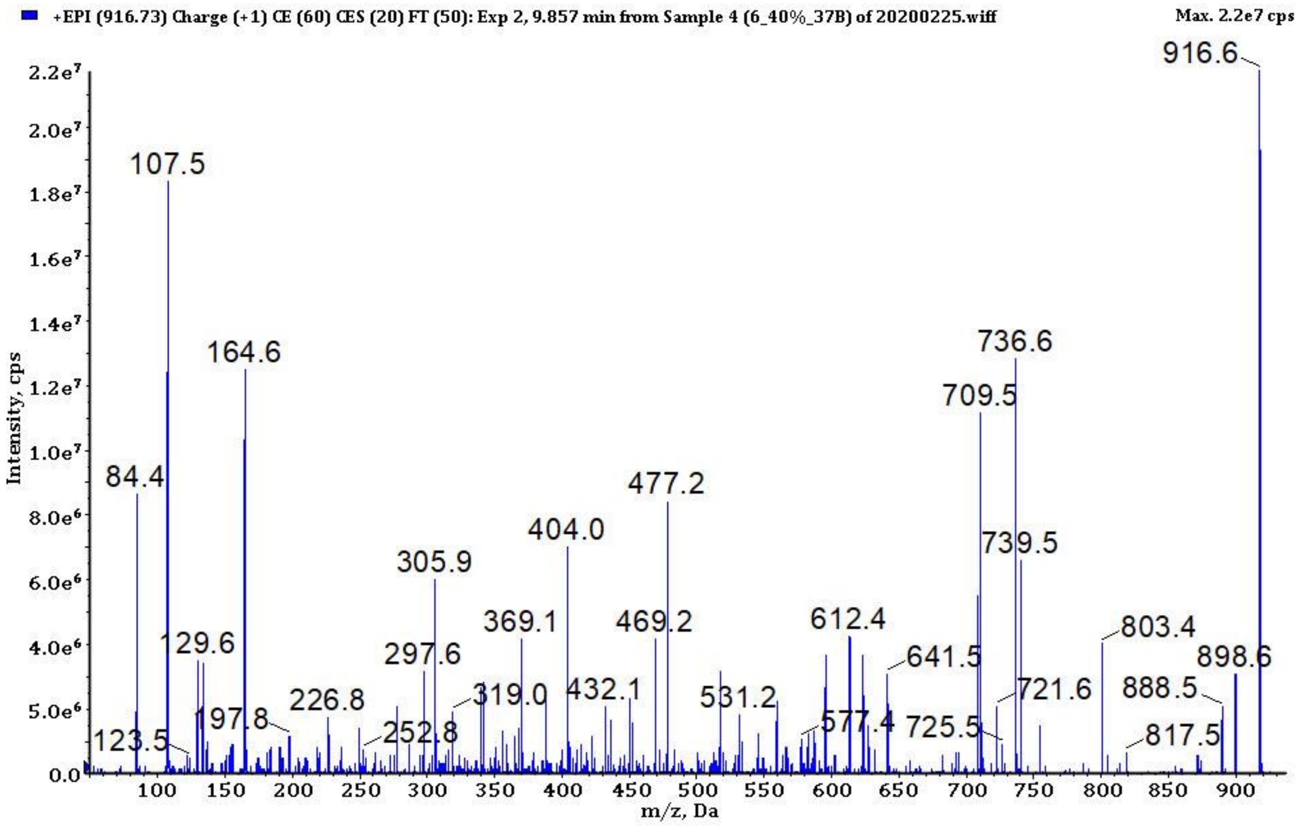

Figure S11. The enhanced product ion mass spectrum of anabaenopeptin AP915 with the suggested structure Tyr + CO – [Lys + Val + HTyr + MeHTyr + Ile] and the following fragment ions: *m/z* 916 $[M + H]^+$, 898 $[M + H – H_2O]^+$, 888 $[M + H – CO]^+$, 817 $[M + H – Val]^+$, 803 $[M + H – Ile]^+$, 739 $[M + H – HTyr]^+$, 725 $[M + H – MeHTyr]^+$, 721 $[M + H – HTyr – H_2O]^+$, 725 $[M + H – MeHTyr]^+$, 709 $[M + H – (CO + Tyr)]^+$, 612 $[M + H – (MeHTyr + Ile)]^+$, 477 $[M + H – Tyr – (HTyr + Val)]^+$, 369 $[MeHTyr + HTyr + H]^+$, 305 $[MeHTyr + Ile + H]^+$, 164 MeHTyr immonium, 84 Lys.

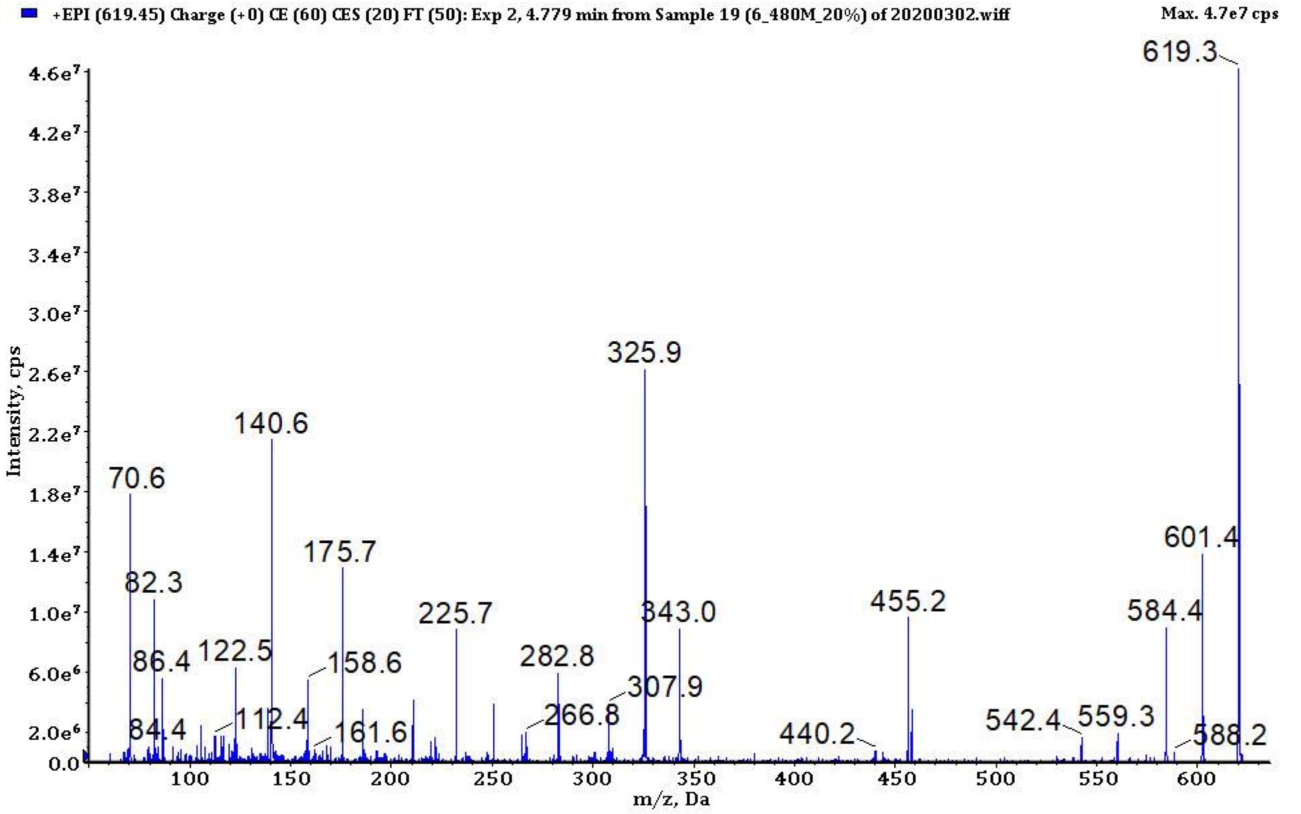

Figure S12. The enhanced product ion mass spectrum of aeruginosin AER618 with the suggested structure Hpla + Leu/Ile + Choi + Arg and the following fragment ions: *m/z* 619 $[M + H]^+$, 601 $[M + H – H_2O]^+$, 559 $[M + H – H_2O – CH_2N_2]^+$, 455 $[M + H – Hpla]^+$, 325 $[Choi + Arg + H – NH_3]^+$, 307 $[Choi + Arg + H – NH_3 – H_2O]^+$, 282 $[Choi + Arg + H – CH_2N_2 – H_2O]^+$, 70 and 175 Arg, 122 and 140 Choi ions, 86 Leu/Ile immonium.
